# S Phase Progression in Human Cells Is Dictated by the Genetic Continuity of DNA Foci

**DOI:** 10.1371/journal.pgen.1000900

**Published:** 2010-04-08

**Authors:** Apolinar Maya-Mendoza, Pedro Olivares-Chauvet, Alex Shaw, Dean A. Jackson

**Affiliations:** Faculty of Life Sciences, University of Manchester, Manchester, United Kingdom; Massachusetts General Hospital, Howard Hughes Medical Institute, United States of America

## Abstract

DNA synthesis must be performed with extreme precision to maintain genomic integrity. In mammalian cells, different genomic regions are replicated at defined times, perhaps to preserve epigenetic information and cell differentiation status. However, the molecular principles that define this S phase program are unknown. By analyzing replication foci within discrete chromosome territories during interphase, we show that foci which are active during consecutive intervals of S phase are maintained as spatially adjacent neighbors throughout the cell cycle. Using extended DNA fibers, we demonstrate that this spatial continuity of replication foci correlates with the genetic continuity of adjacent replicon clusters along chromosomes. Finally, we used bioinformatic tools to compare the structure of DNA foci with DNA domains that are seen to replicate during discrete time intervals of S phase using genome-wide strategies. Data presented show that a major mechanism of S phase progression involves the sequential synthesis of regions of the genome because of their genetic continuity along the chromosomal fiber.

## Author Summary

Eukaryotic DNA synthesis is regulated with exquisite precision so that genomes are replicated exactly once before cell division occurs. In simple eukaryotes, chromosomal loci are preferentially replicated at specific times of S phase, in part because of their differential sensitivity to cell cycle regulators and in part as a result of random choice. Mammals, with ∼250-fold larger genomes, have more complex replication programs, within which different classes of chromatin replicate at defined times. While the basic regulatory mechanisms in higher eukaryotes are conserved, it is unclear how their much more complex timing program is maintained. We use replication precursor analogues, which can be visualized in living or fixed cells, to monitor the spatial relationship of DNA domains that are replicated at different times of S phase. Analyzing individual chromosome, we show that a major mechanism regulating transitions in the S phase timing program involves the sequential activation of replication domains based on their genetic continuity. Our analysis of the mechanism of S phase progression in single cells provides an alternative to genome-wide strategies, which define patterns of replication using cell populations. In combination, these complimentary strategies provide fundamental insight into the mechanisms of S phase timing in mammalian cells.

## Introduction

DNA synthesis in eukaryotes must be performed with absolute precision as any defects compromise genetic integrity. In all eukaryotes, DNA is duplicated during S phase of the cell cycle, which is regulated to ensure that DNA synthesis is completed before mitosis can begin [1]–[3]. During synthesis, different regions of the genome are replicated at specific times [4]–[6], perhaps as a part of a fundamental mechanism that ensures the preservation of epigenetic information [7]. Within this timing program, chromatin within gene-rich chromosomal R-bands is known to begin early in S phase, before synthesis of heterochromatic G-bands takes place. This general structure can be revealed at low resolution, using cytological chromosome banding [8], and at higher resolution using genome-wide strategies [9]–[15].

Recent developments in genome-wide analysis have revolutionized our ability to define the structure of S phase in higher eukaryotes. However, detailed analysis of the replication program has been limited by our understanding of the molecular mechanisms that control how specific origins are used at different times. In mammalian cells, recent studies have shown that local chromatin environments define a general preference for origins that are activated during early S-phase [10]–[15]. Regions that engage synthesis at the onset of S phase frequently have a locally high gene density and correspondingly high levels of RNA synthesis. In addition, more detailed analysis is beginning to explore how local chromatin features such as the distribution of CpG islands [14] and local chromatin accessibility [15] contribute to patterns of origin selection.

Single cell studies provide an alternative strategy for understanding S phase progression. Active sites of DNA synthesis can be revealed as replication foci [16],[17], which contain groups of replicons that are replicated together within dedicated replication factories [18]; such replicon clusters typically contain 3–5 replicons within ∼1 Mbp of DNA [19],[20]. DNA foci are thought to represent fundamental unit of chromosome structure [19]–[23] that are defined by local chromatin environments [23]–[25] and replicated during defined intervals of S phase [26],[27]. Perhaps importantly, foci that are replicated during consecutive intervals of S phase appear to lie side-by-side in nuclei [28],[29], suggesting that their organization contributes to replication timing.

During S phase, the organization of replicons within replicon clusters defines how long individual DNA foci are engaged in synthesis. In HeLa cells, during early S phase, the average speed of fork elongation is ∼1.5 kbp/min/fork [19],[30]. As the average distance between adjacent origins in replicon clusters is ∼150 kb (90% of adjacent origins are typically ∼50–250 kb apart) most will be engaged in synthesis for 1–2 h before the internal forks from adjacent replicons meet and terminate by fork fusion. When this occurs, the rate of synthesis can only be maintained if new origins are activated. Hence the progressive activation and completion of synthesis within the ∼1 Mbp DNA foci defines a replication timing program within which different cohorts of foci are replicated within time zones that occupy ∼1–2 hours of S phase.

**Figure 1:**
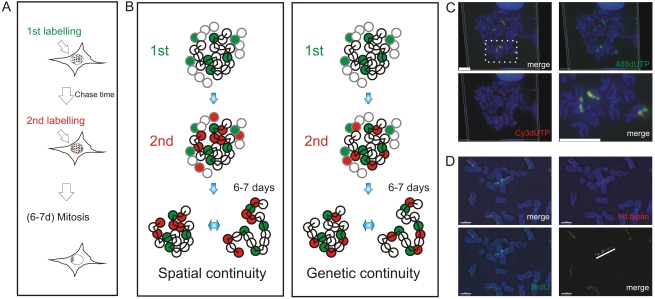
Double-labeled replication foci are segregated in specific regions of mitotic chromosomes. Different dUTP analogues were incorporated into newly replicated DNA and individual chromosomes resolved by random mitotic segregation over 6–7 days (A). Different models (B) show possible relationships between individual DNA foci that are replicated at different times of S phase. In each panel, the replication foci of a single CT (spheres with black rims) and parts of three adjacent CTs (spheres with grey rims) are shown. Foci within the central CT are genetically linked along the chromosome fiber (black zig-zag line). During pulse labeling, some foci are labeled during the 1^st^ pulse (green) and others during the 2^nd^ (red). At this time, the alternative models are indistinguishable, with all green foci lying adjacent to neighboring red foci. 6–7 days later, the foci of individual CTs can be visualized as the surrounding CTs are no longer labeled. The innate plasticity of CTs (2 inter-changeable forms are shown) supports distinct predictions about S phase progression: i) if progression is based on spatial continuity of foci at the time of labeling subsequent changes in CT structure will degrade the side-by-side relationship of foci whereas ii) if progression is based on genetic continuity the side-by-side relationship will be preserved. HeLa cells (C) were labeled with AF448-dUTP (green) and Cy3-dUTP (red), grown for 6 days and DAPI-stained chromosome spreads prepared. Deconvolution microscopy shows that 100% (n = 65 chromosomes from 25 metaphase plates) of the labeled chromosomes incorporated both dUTP analogues and that all labeled regions (note that labeling appears in chromosomal bands at this level of resolution) contained both analogues. A merge of the individual channels and a high-resolution merge of the highlighted region (rectangle) are shown to emphasise co-association of the 1^st^ and 2^nd^ labels. Diploid human fibroblasts (D) were labeled with biotin-dUTP and BrdU with an intervening unlabeled period of 1h. Labeled chromosomes were resolved by random mitotic segregation (6 days) and confocal imaging performed following indirect immuno-fluorescence using specific antibodies to biotin (red) and BrdU (green). Individual red and green channels and a channel merge were overlaid on the DAPI-stained chromosomes as shown. Merged images with the DAPI removed (D, bottom right panel) were used to demonstrate the co-association of foci along individual chromosomes - the white line highlights the labeled foci along one chromatid of a single chromosome. Scale bars: 10 µm in (C) and 5 µm in (D).

Mechanisms of origin selection that define S phase timing are known to show remarkable plasticity during cell differentiation [10],[12],[15]. However, within a particular cell type, the extent to which DNA replication is deterministic – and hardwired by chromosome structure – or stochastic – and so varies for cell to cell – remains a matter of debate. To address this question, we designed an experimental approach that would allow us to analyze the spread of DNA synthesis throughout nuclei of individual cells (Figure 1). Sites of DNA synthesis within DNA foci were labeled with thymidine analogues using pulse and pulse-chase-pulse strategies and analyzed over many days. Initially, labeled foci are distributed throughout all chromosomes but as cells proliferate random mitotic segregation reduces the number of labeled chromosomes within individual cells so that chromosome territories (CT) and their DNA foci are resolved. Immediately after labeling it is impossible to establish the extent to which adjacent foci are related by their spatial and/or genetic continuity, as the alternative models are indistinguishable. However, following chromosome segregation, the plasticity of CT structure [24] allows the spatially and genetically determined models to be distinguished (Figure 1B). Hence, over many cell division cycles, the analysis of individual CTs provides a high-resolution memory of cis- and trans-activation events that define the replication timing program.

We used 3D and 4D light microscopy to analyze the organization of DNA foci within individual CTs of nuclei and mitotic chromosomes. We show that the sequential replication of DNA foci is defined by their genetic association along individual chromosomes. To visualize the genetic association directly, we analyzed individual DNA fibers from cells that were labeled during sequential 1 h intervals of S phase. We conclude that the sequential activation of adjacent replicon clusters represents a major mechanism of S phase progression. Indeed, once early synthesis has begun, only a minority – about 10% - of *de novo* initiation events are genetically uncoupled from sites that were engaged in synthesis earlier during S phase. Finally, in order to integrate this conclusion with the analysis of replication using genome-wide strategies, we used bioinformatic tools to show that the structure of replicon clusters within DNA foci and lengths of replication timing domains correlate with extremely high significance. This is consistent with DNA foci being the stable higher-order units of chromatin packaging that define the replication timing program in mammalian cells.

## Results

### S phase progression is defined by the spatial organization of DNA foci

In HeLa cells in early S phase, the template for DNA synthesis is folded into DNA foci that can be labeled with a variety of modified thymidine analogues and visualized in both living and fixed cells (Figure S1). Different pulse and pulse-chase-pulse strategies can then be used to evaluate the relationship of foci that are engaged in DNA synthesis during different intervals of S phase (Figure S2). In mid/late S phase, the spatial relationship of foci that were labeled during consecutive intervals of S phase is evident because distinct patterns of active sites are seen at this time (Figure S1A and S1C). In early S phase (Figure S1B), in contrast, spatial analysis at the time of labeling is much less informative because of the high density of active sites.

**Figure 2:**
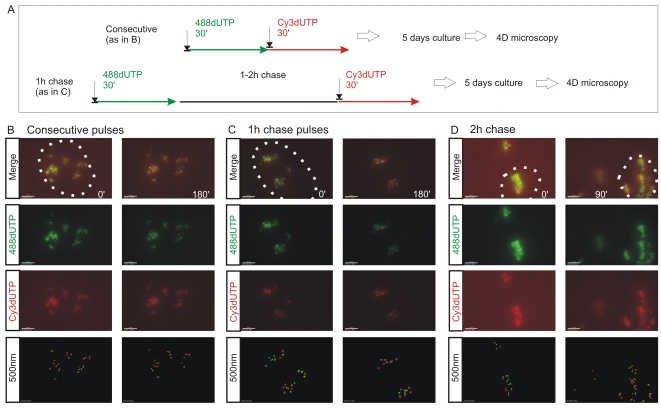
The spatial architecture of DNA foci is maintained in living cells. 4D time-lapse imaging was used to monitor the dynamic behavior of DNA foci (A–D). HeLa cells were labeled with consecutive pulses of AF488-dUTP (green) and Cy3-dUTP (red) with different times of intervening chase (A) and individual CTs resolved by mitotic segregation (B–D). Using consecutive pulses with no intervening unlabeled period (B), all CTs were labeled with both precursors, which were also co-associated within sub-regions of individual CTs (B) (Video S1, S2). CTs are seen to be highly dynamic, yet despite changes resulting from cell movement the spatial co-association of 1^st^ and 2^nd^ pulse-labels was always maintained throughout the imaging time course. Clear spatial co-association of the 1^st^ and 2^nd^ pulses was also seen when pulses were separated by unlabeled chase periods of 1h (C) (Video S3) and 2h (D), with adjacent foci labeled during the 1^st^ and 2^nd^ pulses maintaining separations of ∼500 nm (B, 1 h chase: 390+/−148 nm n = 53; D, 2 h chase: 438+/−141 nm n = 57). For each labeling program (B–D), typical examples show isolated CTs within individual cells (nuclei are marked by dotted lines) that were imaged at 15 min intervals using time-lapse 3D microscopy for 3 h or more (data not shown). Individual green and red channels together with a two channel overlay (merge) and centers of mass of foci labeled in red and green channels (500 nm: labeled sites are depicted by foci of 500 nm diameter) are shown (B–D). For each experiment (B–D), 2 time points (0 and 90 or 180 min) are shown to emphasise changes in the structure of foci within individual CTs over time. Scale bars: 4 µm.

**Figure 3:**
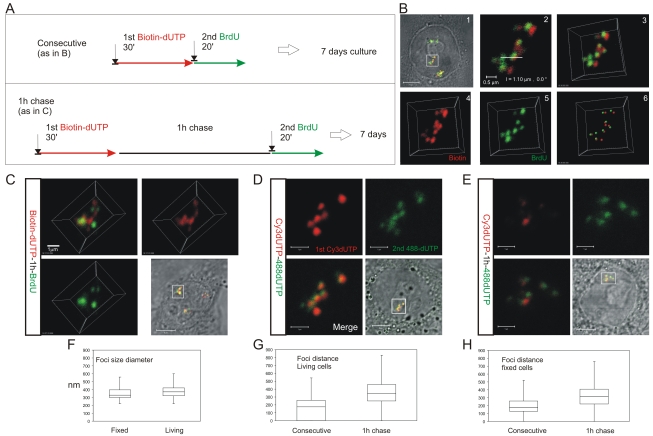
The S phase program is defined by the temporal activation of DNA foci at adjacent positions within CTs. HeLa cells were labeled with consecutive pulses of biotin-dUTP and BrdU either without or with an intervening unlabeled chase and grown for 6–7 days to resolve labeled CTs (A). Cells with individual labeled CTs were analyzed by confocal microscopy (B). Following consecutive pulses of biotin-dUTP (red) and BrdU (green) a cell with 3 CTs was selected (B1) and confocal sections of an individual CT (boxed area) taken (B2 shows a single confocal section) to produce a 3D projection of the entire CT (B3). Individual channels from the 3D projection were separated (B4,5) and mass centers of the labeled foci defined and combined (B6). Within this CT most foci are double-labeled though some are only labeled with the 1^st^ or 2^nd^ precursor. Double labeled CTs were analyzed following consecutive pulses (B,D) or pulses with an intervening chase (C,E) to monitor spatial continuity during S phase progression. Equivalent cells were analyzed by indirect immuno-fluorescence in fixed cells (B,C), after replication with biotin-dUTP (red) and BrdU (green), or under live imaging conditions (D,E), after replication with AF488-dUTP (green) and Cy3-dUTP (red). (D–E) show 2D confocal sections and (C) a 3D maximum projection of CTs highlighted (square) in the corresponding phase contrast images. Individual foci were measured to define their size distribution (F; diameter of foci; n = 60). Nearest neighbor analysis of labeled foci was performed (G,H) to define the separation of adjacent foci labeled with the 1^st^ and 2^nd^ analogues during consecutive pulses and pulses with an intervening chase. In both living (G; n = 200; t test: p<2.05E-31) and fixed (H; n = 167; t test: p<4.7E-18) cells the labeling patterns differed with a high degree of statistical significance. Scale bars: 5 and 0.5 µm in (B) and 5 and 1 µm in (C,D), respectively.

To evaluate the alternative models of S phase progression described in Figure 1, cells were labeled with two consecutive pulse-labels and grown for many days to leave ∼3 labeled CTs/cell (Figure 1C, Figure 2, Figure 3). As a control, we first monitored the co-association of labeled foci in metaphase, as this defines their distribution within individual chromosomes (Figure 1). Metaphase images, from cells that were labeled during early S phase, showed that all labeled chromosomes within double-labeled cells contained early S phase foci that incorporated both the 1^st^ and 2^nd^ precursors. However, as chromosome condensation during metaphase limits the resolution of the spatial analysis, we next monitored the level of co-association within interphase CTs [23]. Analysis of CTs showed that foci labeled with the 1^st^ replication precursor were within 500 nm of a focus labeled with the 2^nd^. In addition, time-lapse imaging of foci in living cells showed this co-association to be maintained when cells were monitored for up to 3 hours. Throughout the imaging time course (Figure 2 and Video S1, S2, S3), individual CTs showed dramatic plasticity [31], with shape transformations during cell movement resulting in early S phase foci displaying frequent relative positional shifts of 0.2–0.6 µm over 30 min. Notably, during these shifts, the association of adjacent foci labeled during the 1^st^ and 2^nd^ pulses was always maintained (25 CTs were analyzed by live imaging and labeled foci showed the same behavior in all cases).

### The sequential activation of DNA foci is defined by their spatial continuity within individual chromosomes

To reinforce the interpretation of time-lapse imaging, we evaluated the spatial relationship of interphase foci labeled during consecutive intervals of early S phase using 3D confocal microscopy (Figure 3). To test the models described in Figure 1, we measured the spatial separation of nearest foci containing the 1^st^ and 2^nd^ precursors using both consecutive pulses and pulses separated by intervening unlabeled periods of 1 or 2 hours. Experiments were performed using both fixed (Figure 3B and 3C) and living (Figure 3D and 3E) cells. Living cells were analyzed directly and fixed cells were processed for 3D confocal imaging by indirect immuno-labeling.

Following image capture, image analysis software was used to define the center of mass of labeled sites (Figure 3B6) and then measure the 3D separation of the nearest sites labeled during the 1^st^ and 2^nd^ pulses (Figure 3G and 3H). Under the experimental conditions used, DNA foci in HeLa cells have an average diameter of ∼350 nm (Figure 3F). Moreover, as living and fixed cells show the same size distribution, our experimental strategies do not appear to disrupt local chromatin architecture during processing and imaging (Figure 3F; t test association probability p<0.07 n = 60). While analysis of both fixed and living cells demonstrates the stability of foci with sizes of 300–500 nm, we note that recent advances in light microscopy (3D-SIM and SMI microscopy) reveal that individual foci can be resolved into sub-domains with an average size of ∼125 nm [Cristina Cardoso and Vadim Chagin, Technische Universität Darmstadt, personal communication].

When unsynchronized cells were labeled with consecutive pulses, most foci were labeled with both precursors (Figure 3B and 3D); as synthesis within individual foci is not synchronized, a minority of foci might be labeled with only one precursor because they began or completed synthesis during the 1^st^ or 2^nd^ labeling periods. However, when the pulses were separated by 1 hour (Figure 3C and 3E) ∼50% of foci were labeled with only one analogue (43.5% of foci in living cells (n = 200) and 52% in fixed cells (n = 146)). Nearest neighbor analysis was used to explore this spatial relationship quantitatively (Figure 3G and 3H). With consecutive pulses, the average center-center separation of the nearest red and green labeled sites was ∼150 nm (Figure 3B and 3D) – as most foci are double labeled this center-to-center separation is less than the average diameter of individual foci. With an intervening chase of 1 h, the separation between adjacent foci labeled during the 1^st^ and 2^nd^ pulses increased to ∼350 nm (Figure 3G and 3H). As this center-to-center separation is similar to the average diameter of foci in early S phase (Figure 3F) foci labeled during the consecutive intervals of S phase must lie close to or touching their nearest neighbor.

Two important controls emphasize the significance of this nearest neighbor analysis. First, we analyzed individual foci that were labeled simultaneously with 2 replication precursor analogues (Figure S3). This defines the reliability of distance measurements and the effect of experimental noise on the precision of data generated by the analysis. To demonstrate a worst-case-scenario, red and green foci with >2-fold average intensities were seen to give an average separation of no more than 75 nm (Figure S3). Second, we also measured the separation of foci labeled during either 1^st^ or 2^nd^ pulse to define the distribution of foci that were labeled with each precursor. Under all labeling conditions used, the average separation of nearest early S phase foci was ∼500 nm (Figure S4), which is highly significantly different to the separation of neighboring foci labeled by consecutive pulses with an intervening chase (t test = 2.955E-12 comparing separation of BrdU foci in Figure S4D with separation of biotin and BrdU foci in Figure 3H).

In a parallel study, we also performed a nearest neighbor analysis using normal diploid human fibroblasts (MRC5; Figure S5). While these diploid cells appear to have slightly larger early foci (513+/−116 nm; n = 200) than HeLa cells, perhaps as a consequence of their flattened shape, foci labeled with a separation of 1 h nevertheless maintained a strict side-by-side relationship (separation was 556+/−114nm; n = 155). These experiments show that DNA foci labeled during consecutive intervals of S phase retain a nearest neighbor relationship independently of changes in CT structure, consistent with the spatial relationship at the time of labeling being defined by the genetic connectivity of DNA foci along chromosomes. The significance of this strict side-by-side relationship was reiterated using *in silico* simulations to model the activation of DNA foci (Figure S6).

### The replication timing program correlates with the spatial context of DNA foci

We next attempted to reinforce the links between S phase progression and the genetic continuity of DNA foci by monitoring the distribution of foci labeled during widely separated intervals of S phase. First, we analyzed cells in early S phase after labeling replication foci with 3 sequential replication precursors each separated by 1 hour (Figure S7). As expected, the separation of both consecutive labels – the separation between the 1^st^–2^nd^ and 2^nd^–3^rd^ precursors - was ∼350 nm (Figure S7). However, a significantly larger separation of ∼500 nm was seen when the separations of sites labeled with the 1^st^ and 3^rd^ precursors was measured (Figure S7C). This shows that even though the folding of DNA foci within individual CTs is complex and dynamic (Figure 2) the foci labeled at different times of early S phase show a progressive separation over time.

This progressive synthesis of early S phase replication foci is consistent with synthesis spreading along chromosomes at a rate of ∼200 nm/h. Over longer periods - with separations of >4 hours - the linear continuity of labeled sites is difficult to define because nearest neighbor relationship are degraded by chromosome folding (Figure S8) and the distribution of euchromatin and heterochromatin in CTs [17],[24],[25]. Based on this observation, we would not rule out the possibility that early and mid/late S phase have distinct characteristics. Towards the end of early S phase, as the replication of euchromatin completes, many forks appear to pass from the early to mid/late replication domains [10]–[12]. At this time of S phase, a significant fraction (∼5%) of chromatin is replicated by forks that extend for at least 500 kbp. Such temporal transition regions in the replication program [10] apparently engage synthesis for many hours without encountering and activating potential origins in heterochromatin.

### Visualizing replication domains on single DNA molecules defines the genetic contribution to S phase progression

**Figure 4:**
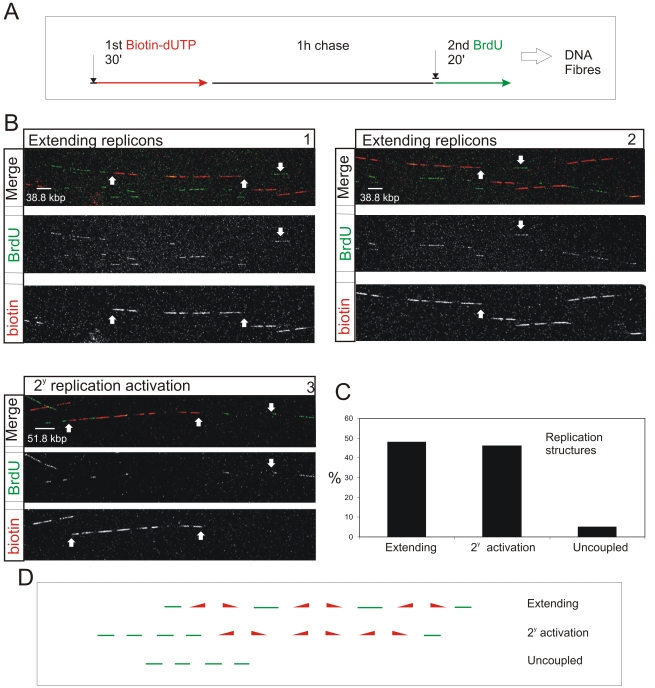
S phase progression correlates with the sequential activation of replicon clusters as defined by their genetic continuity along individual chromosome. Cells were pulse-labeled with biotin-dUTP and BrdU separated by 1 h without label (A) and double-labeled DNA fibers of >0.8 Mbp in length collected (B). Typical examples (B) show two major classes, where the 1^st^ and 2^nd^ pulse labels were incorporated into genetically adjacent replicon clusters. (B) panels 1–2 show a single fiber that extends over two adjacent imaging fields; the up pointing arrows show part of the replicon cluster labeled with biotin-dUTP during the 1^st^ pulse; down pointing arrows show BrdU incorporation between two growing replication forks. Panel 3 shows a typical cluster with four active replicons, which were labeled during the 1^st^ pulse, and two adjacent replicon clusters (defined my multiple Br-labeled tracks) activated during the 2^nd^ pulse. In other clusters the labeling was confined within a single active cluster that was labeled during both periods of incorporation (Figure S9). To analyze genetic continuity, BrdU incorporation was monitored in the vicinity of stretches of biotin labeled DNA of >0.8 Mbp DNA with labeling properties expected for early S phase replicon clusters (B; n = 50). Double labeled fibers were scored in two classes (C,D): 1) Extending replicons - contained biotin-labeled replicons with internal forks labeled with BrdU during the 2^nd^ pulse. 2) Clusters with secondary activation - contained multiple BrdU patches in the DNA fiber adjacent to the biotin-labeled cluster. In the same spread fields, fibers containing tracks labeled uniquely with BrdU (ie >250kbp from biotin-labeled tracks; D) were also recorded (C). The sizes of scale bars are shown on individual panels.

Nearest neighbor analysis is consistent with a genetically defined next-in-line model, which operates in cis within individual CTs (Figure 1). We next wanted to evaluate the extent to which this cis activation defines S phase progression. In nuclei, however, analysis is compromised by the dynamic properties of DNA foci within individual CTs. To avoid this limitation, we analyzed the genetic relationships of replication pulses along individual DNA fibers (Figure 4). DNA fibers were prepared by direct spreading of cells labeled with biotin-dUTP and BrdU with an intervening 1 h chase. Spreads were prepared directly from cells without prior DNA isolation in order to image isolated ∼1–2 Mbp DNA fibers. As careful spreading, with only ∼5 labeled cells per spread, prevents mixing of fibers from individual labeled cells [32], this approach allows us to capture biotin-labeled fibers from cells that were engaged in DNA synthesis during the 1^st^ labeling period. Regions of spreads with dispersed biotin-labeled fibers were located and randomly selected fields recorded; low magnification was used so that each imaging field contained fibers with at least 0.8 Mbp of DNA. In 144 fields, from 4 equivalent experiments, the fibers analyzed contained 450 Mbp of DNA in total.

Double-labeled fibers were analyzed, as any forks growing throughout the labeling period will incorporate both 1^st^ and 2^nd^ precursors, which will be separated by a predictable distance that reflects the rate of fork elongation (Figure 4A and Figure S9). As seen before [19], the active replicons are often clustered into small groups that typically contain ∼0.5–1 Mbp of DNA. This clustering is exemplified by the DNA fibers shown in Figure 4B. The first example (Panels 1 and 2) shows two adjacent imaging fields that contain a single fiber of >1.5 Mbp. This fiber has 3 replicons in the center and 2 on the right that were active during the 1^st^ pulse (biotin in red). These replicons are linked genetically as replication in the DNA between them is completed during the 2^nd^ pulse (BrdU in green). On the left of the same fiber, three patches are labeled during the 2^nd^ pulse, showing that replicons in the adjacent DNA are activated during the 2^nd^ labeling period. The short cluster shown in panel 3 contains 4 active replicons with an average size of 90 kbp. In this particular example, secondary origins are activated in replicons on both sides of the central cluster during the 2^nd^ labeling period.

Using fibers like those shown (Figure 4B), two distinct classes of double-labeled fiber were scored, based on labeling within the proximal flanking DNA (Figure 4C and 4D). Replicon clusters with ‘extending’ forks were scored when replicons labeled during the 1^st^ pulse were flanked by single DNA tracks labeled during the 2^nd^ pulse, consistent with continued elongation of the out-growing forks from the flanking replicons of the primary cluster. Replicon clusters with ‘secondary activation’ were scored when DNA flanking the primary cluster also contained multiple tracks labeled during the 2^nd^ pulse, which is only possible if additional forks are activated within the flanking DNA. The structure of replicons within the primary (biotin-labeled) clusters defines the frequency of these two populations (Figure S9). Notably, clusters with extending forks had widely dispersed origins (∼200 kbp apart on average) whereas clusters with secondary initiations within the flanking DNA had shorter inter-origin distances (∼125 kbp apart on average). This difference presumably reflects the temporal relationship between the completion and activation of synthesis in adjacent replicon clusters.

Preparation and staining of DNA fibres that contain >1 Mbp of DNA is technically challenging. However, the use of quality controls to monitor spreading and measurement of the labeled tracks (Figure S9) ensure reliability of the data generated. In all of the scored fibres, the separation of the biotin- and BrdU-labeled tracks was consistent with fork elongation rates within the normally accepted range for early S phase of 1–2 kbp/min (Figure S9). In these fibers, the continuity of the labeled tracks demonstrates that the underlying DNA strand must be intact throughout the labeled region.

To complete this analysis, we recorded single-labeled regions in order to define *de novo* initiation events that were remote from previously active replicons and thus ‘uncoupled’ (Figure 4C and 4D) from synthesis during the 1^st^ labeling period. In the random fields used in this analysis, only 5% of labeled tracks were seen to be uniquely BrdU-labeled (Figure 4C). These observations suggest that genetically adjacent DNA foci are replicated during consecutive intervals of S phase. This genetic spread of synthesis appears to be a major mechanisms, as while the stochastic activation of potential origins is not precluded, remote initiation events, which are uncoupled from previously active replication foci, account for no more than 10% of initiation events once S phase has begun.

### Individual DNA foci correlate with genome-wide replication timing domains

**Figure 5:**
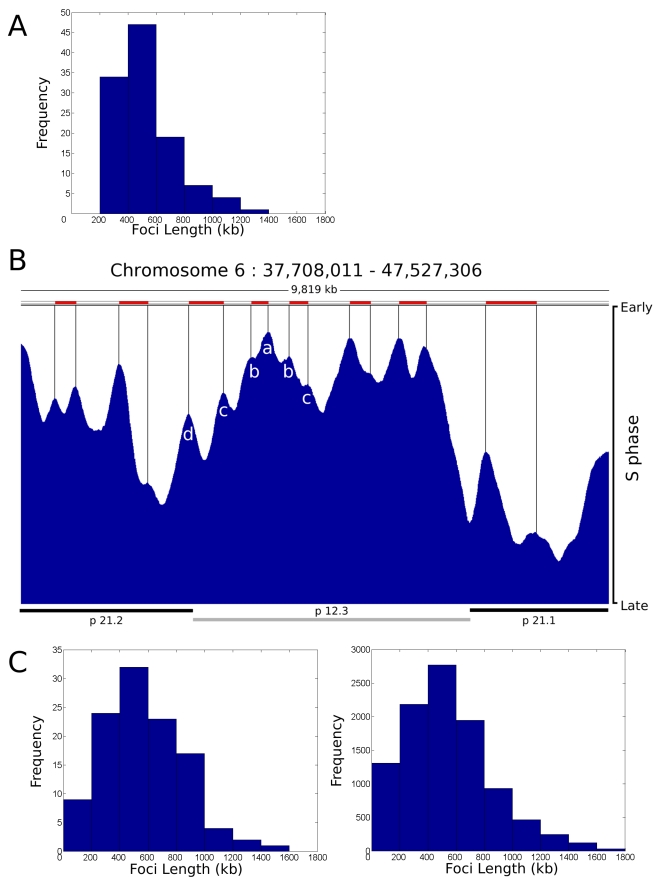
Replication timing domains correlate with DNA foci. A distribution profile for the length of replication timing domains was generated (A) using randomly selected regions of human chromosome 6 (n = 112, representing 59 Mbp (35%) of ch6), using data from [10]. Points of inflection in the timing profile were used to define replication timing domains – peaks corresponding to 6 such domains are identified in the center of the region shown (peaks a–d in B). The typical region shown (B) contains 1 central chromosomal R-band (light grey bar below) flanked by two G-bands. The G-band on the left is cytologically light staining and replicates during early S phase whereas as the one on the right is dark staining and replicates late in S phase. Domains in R- and G-bands were analysed separately, but as no significant difference was seen a composite genome-wide profile was generated. Distribution profiles for the length of DNA in individual DNA foci were also generated using data from [19]. Data derived from the profiles was as follows: (A) Mean length, 529.5+/−208.0 kbp, 90% data within 274.7–934.6 kbp; (C) right, simulation for 112 clusters – Mean length, 527.9+/−312.2 kbp, 90% data within 125.7–1,055.2 kbp; (C) left, simulation for 10,000 clusters – Mean length, 549.0+/−306.2 kbp, 90% data within 140.4–1,144.0 kbp. Correlation Coefficients for each pair of profiles were as follows: A:C_112_ = 0.9193; A:C_10000_ = 0.9100; C_112_:C_10000_ = 0.9820.

During our analysis of replication foci within individual cells we deliberately used a holistic approach in order to avoid any bias that might arise if specific genomic regions were targeted for analysis. To validate our conclusions, we next attempted to integrate the single cell data (Figure 1, Figure 2, Figure 3, Figure 4) with genome-wide data sets [10]–[15], which define the average pattern of synthesis across cell populations. To compare the structure of genome-wide timing domains with replication foci, we first defined the distribution profile of replication timing domains on selected regions of a specific human chromosome (Figure 5A) using genome-wide data sets taken from Desprat et al. [10]. Randomly selected regions of human chromosome 6 with ∼10 Mbp of DNA (1 region is shown in Figure 5B) were sampled and points of inflection in the data readout used to define peaks in the timing profile. Individual peaks represent domains of discrete replication timing and peak heights (Figure 5B) define the average time of replication of the domain across the cell population analyzed – the highest peaks are replicated predominantly at the onset of S phase. When replication domains from different regions of chromosome 6 were combined the resulting distribution profile (Figure 5A) showed the average domain to contain 529.5+/−208.0 kbp of DNA.

For comparison with the timing data, we generated a series of distribution profiles that simulate the DNA content of populations of DNA foci in human cells. Profiles were generated using published data [19] that describes the distribution of replicon sizes and the number of replicons/cluster in human HeLa cells. In the two distribution profiles shown (Figure 5C) the first describes a typical profile for a population of 112 DNA foci – for direct comparison with the data set in Figure 5A – and the second shows the profile for a much larger sample. With average DNA contents of 527.9+/−312.2 kbp and 549.0+/−306.2 kbp of DNA, respectively, these simulations show that the DNA contents of replication timing domains and DNA foci have a high degree of similarity, with correlation coefficients in excess of 0.9 (Figure 5).

Figure 5 also shows the timing relationship between adjacent replication domains using genome-wide analysis of cell populations. The early replicating band p12.3 shows an example of how replication proceeds across a chromosomal domain, which in this typical example contains ∼5 Mbp of DNA. At the left side of this region, 6 timing domains (seen as peaks on the timing profile) are clearly structured so that the central region (Figure 5B, region a) is replicated at the onset of S phase and the adjacent flanking regions (Figure 5B, regions b–d) are replicated sequentially as S phase proceeds. While the structure of peaks and valleys in the timing profile shows that individual cells in the population activate replication of the respective domains at slightly different times, the general trend is clearly consistent with the sequential activation of genetically adjacent timing domains across this region of chromosome 6 in human ES cells.

This comparison highlights a number of fundamental features of chromatin organization that define the efficacy of DNA replication. Most importantly, it is clear that the amount of DNA within both DNA foci and replication timing domains is dramatically different from the average size of individual replicons, which typically contain 100–150 kbp of DNA in human cells [19],[20]. This implies that the replication timing domains must contain groups of replicons that are replicated together. In addition, if individual timing domains were single replicons it would only be possible to duplicate 1.5×10^9^ bp or ∼25% of their DNA in an S phase of 10 hours, given that synthesis during S phase of a diploid mammalian cells involves ∼750 replication sites at any time [16]–[23]. Hence, the co-replication of replicons clusters within replication timing domains is necessary to complete synthesis on schedule.

While the evidence for replication timing domains that contain multiple replicons is overwhelming, it is notable that individual replicons are not evident at the resolution provided by genome-wide analysis (Figure 5B). This is likely to reflect the redundancy of potential origins, which in human cells are present in ∼10-fold excess relative to actual sites where DNA synthesis initiates [1]–[3],[33]. Features of the local chromatin environment are thought to contribute to origin selection and define the relative efficiency with which different potential origins are used. Even so, origin activation clearly has a strong stochastic component so that different sites are used in different cells (Figure S10). As a result, the timing domains seen in population studies must generate a composite activation profile, which reflects how potential origins are used. The use of different potential origins in different cells will effectively smooth synthesis across chromatin domains so that the distribution of individual replicons is not seen. This means that replicon structure is defined by initiation events within individual cells and that the functional domains that are defined by DNA foci, and not the individual replicons themselves, are the regulatory targets for DNA synthesis.

### The organization of DNA within chromosome territories defines the location of replication factories within the inter-chromatin compartment

The efficacy of a timing program that propagates using the genetic continuity of DNA foci will require that initiation sites that are used at the onset of S phase have an appropriate distribution throughout the genome. Notably, replication foci visualized in metaphase are uniformly spread along chromosomes (Figure 1). While it is not known how this is achieved, genome-wide approaches show that replication will often begin in regions of the genome that are rich in features linked to gene expression [10]–[15]. Interestingly, this conclusion was drawn from single cell studies 15 years ago [34], based on the co-localization of replication factories and active transcription sites at the onset of S phase.

**Figure 6:**
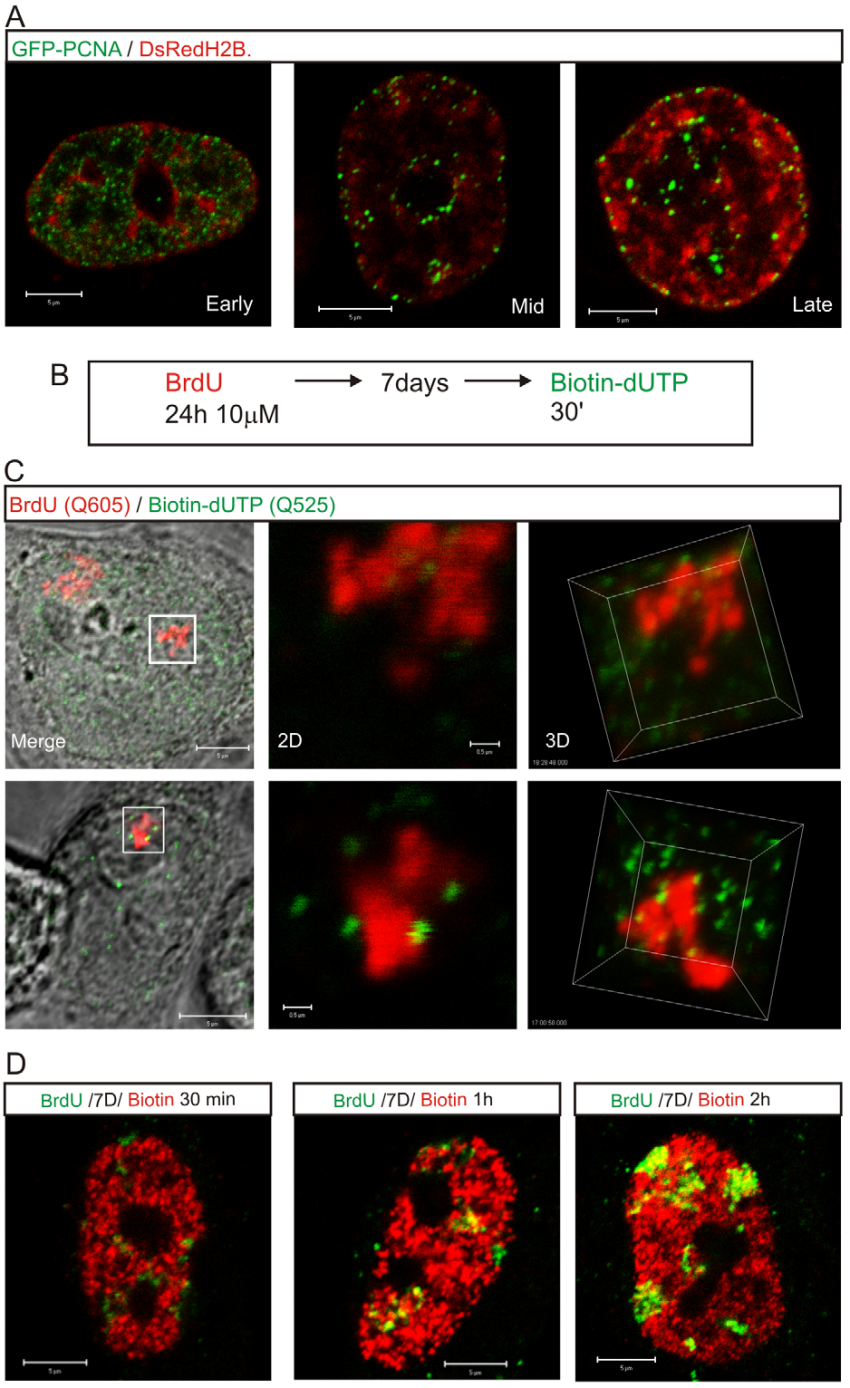
The proximity of DNA foci and the inter-chromatin domain defines the location of sites that are permissive for replication factory assembly. Active sites of DNA synthesis are shown by 3D imaging to be spatially separated from the substrate containing DNA foci (A,C). The distribution of replication factories was monitored using live cell imaging in cells transiently expressing GFP-PCNA (green) and histone H2B-DsRed (red) 24–48 h post-transfection (A). For a high-resolution view (C), entire CTs were labeled with BrdU (red), resolved by mitotic segregation and sites of nascent replication pulse-labeled with biotin-dUTP (green) as shown (B). Labeled sites were visualized using Q-dots and high-resolution images (60 slices with 100 nm Z steps) collected to assess the relative distribution of nascent sites and associated CTs during early (C, top) and mid/late (C, bottom) S phase. Highlighted regions (white boxes) are shown at high magnification in 2D and 3D, as indicated. Using the same labeling program (B) and different chase periods (for biotin labeling: biotin-dUTP is consumed in 10–15 min so longer incubations incorporate the initial labeling pulse followed by an unlabeled chase) co-localization of the BrdU (green) and biotin (red) labels was evaluated in confocal sections of fixed cells by indirect immuno-fluorescence (D) to monitor the location of newly replicated DNA. Following the 2^nd^ pulse, the typical early S phase cell shown had only 11% of voxels in biotin-labeled foci that also contained BrdU. Following 1 and 2 h chase periods the level of co-localization increased to 31% and 59%, respectively, again in the typical early S phase cells shown. Scale bars: 5 and 0.5 µm in panels with individual nuclei and high-magnification, respectively.

Potential origins are thought to be equivalent when they are established well before the onset of S phase [1]–[3]. Hence, origin selection at the beginning of S phase must reflect the local chromatin environment within nuclear domains where replication factories are assembled. In this regard, it is notable that early replication factories are associated with nuclear domains that contain open chromatin whereas replication during mid/late S phase spreads to the chromatin-dense nuclear domains (Figure 6). This is confirmed by the structure of sites that contain nascent DNA (Figure 6A), which are located within the chromatin compartment at the interface between the chromatin and inter-chromatin nuclear domains [22],[23]. During synthesis, the organization of active sites means that DNA foci, which contains the unreplicated template, and the associated factories and nascent product occupy discrete nuclear compartments (Figure 6C). This spatial separation means that during replication of a DNA focus that was labeled with BrdU in an earlier cell cycle the nascent product shows very little immediate co-localization with Br-DNA within the template containing focus. Subsequently, as the nascent chromatin matures, a period of 1–2 h is required before almost complete co-localization is seen (Figure 6D). This arrangement shows how the spatial architecture of the template-containing DNA foci and synthetic factories (Figure 6C) contribute to the dynamic behavior of chromatin during S phase.

## Discussion

Eukaryotic cells have such complex genomes that DNA synthesis must be highly regulated in order to ensure the preservation of genome integrity and epigenetic modifications that define cell type. Surprisingly little is known, however, about the molecular principles by which this is achieved in higher eukaryotes. One key feature of the process, which has been appreciated for many years, is that replication of euchromatin and heterochromatin is structured temporally to occur preferentially during early and mid/late S phase, respectively [8]. This temporal restriction correlates with the differential activity of specific cyclin-CDK complexes [35] and the replication of different classes of chromatin, as defined by post-translational histone modification [36],[37], during early and mid/late S phase.

While the spatial architecture of DNA foci appears to contribute to the structure of the mammalian S phase, the molecular mechanisms involved are not known. To address this question, we designed a single cell strategy to identify molecular links between chromosome organization and the timing of DNA synthesis (Figure 1). Analysis at the level of single cells is based on the structure of DNA foci, which are both functional units of DNA replication and structural units of chromosome organization [17],[22],[23]. The architecture of structural foci within chromosomal sub-domains has been analyzed in numerous recent studies. High-resolution analysis of the distribution of chromatin in domains of 2–10 Mbp has clearly demonstrated that foci typically contain 0.5–1 Mbp of DNA [24],[25],[38]. The most comprehensive study has shown that foci with ∼1 Mbp of DNA are a common feature of genome organization [25] and that foci within transcriptionally active and inactive chromatin domains have distinct properties and nuclear distributions [25]. The spatial architecture of the 1 Mbp DNA domains has been analyzed in detail over length scales ranging from 0.5 to 75 Mbp [39]. Notably, the domains in nuclei are separated in relation to their genetic co-ordinates in the range 0 to 3–5 Mbp but little further separation is seen when sequences are further apart, because of the 3D folding of chromosomes within CTs [39].

### S phase timing is defined by the connectivity of DNA foci

Here, we wanted to assess how higher-order chromatin organization contributes to the S phase timing program in mammalian cells. To do this, we evaluated the relative importance of direct (genetic) and indirect (spatial) chromatin interactions during S phase progression (Figure 1). DNA foci were labeled at different times of S phase and their spatial organization analyzed within individual CTs. Using a nearest neighbor analysis of DNA foci (Figure 1, Figure 2, Figure 3, and Video S1, S2, S3), together with an analysis of labeling continuity on stretched DNA fibers (Figure 4), we show that DNA foci that were labeled during consecutive intervals of S phase maintain a strict spatial co-association over many cell cycles. This demonstrates that foci labeled during consecutive intervals of S phase are genetic neighbors along chromosomes and provides strong evidence that this relationship underlies a ‘next-in-line’ mechanism of S phase progression [28],[29]. Importantly, our experimental design is not directed to specific chromosomal loci or specific times of the cell cycle but instead uses an unbiased and holistic analysis of DNA foci, which are replicated during early S phase; as the labeled foci are not constrained by synthesis at the time of analysis their distribution must reflect a preferred organizational steady state within CTs.

As S phase proceeds, the majority of foci engage synthesis for 1–2 h (Figure S2) before the termination of synthesis by fusion of internal forks is coupled to activation of origins within adjacent DNA foci. The invasion of outgrowing forks into the genetically adjacent foci is one mechanism that in principle could cause structural alterations that allow or stimulate *de novo* origin activation. However, our analysis shows that this is not an inevitable outcome, as some forks grow without encountering conditions where *de novo* origin activation will occur; such regions might have a low density of potential origins [10]–[12]. Forks with these characteristics have been described using both DNA fibers [reviewed in 22] and in recent genome-wide studies [10]–[12], where extended forks of >250 kbp (representing ∼5% of the genome) correlate with the ‘temporal transition regions’ that link replication during early and mid/late S phase. This transition from early to mid/late S phase correlates with a timing transition that can be revealed as a ‘3C-pause’ in DNA synthesis under some conditions of replicative stress [40].

### Genome-wide approaches to map replication timing

Single cell studies and genome-wide analysis of replication in cell populations provide complimentary strategies to explore DNA synthesis. Hence, it is important to understand the strengths and limitations of these strategies and evaluate how key information can be combined to develop a general model of S phase progression. A specific advantage of the genome-wide approach is that replication timing is anchored directly to DNA sequence and annotated features such as chromatin architecture and transcriptional activity. In doing this, genome-wide strategies also provide a composite view of DNA synthesis, which can be interpreted to define the average behavior of cells in the population. Such population approaches have shown that large regions of mammalian genomes are replicated during predictable intervals of S phase and that this generally correlates with features of the chromatin environment, so that highly expressed regions of the genome are replicated early during S phase [10]–[15]. The fact that syntenic regions of the human [9] and mouse [11] genomes replicate at equivalent times implies that this general principle is conserved.

During DNA synthesis, cells must also preserve the epigenetic information in chromatin that defines cell type specific patterns of gene expression. In exploring this aspect of mammalian S phase, genome-wide studies have shown that large genomic regions alter their replication timing when cells are induced to differentiate [10],[12],[15] and that distinct changes in replication timing arise as cells become epigenetically committed to differentiation [41]. Such changes raise obvious questions about mechanisms that link chromatin domains that are selected for synthesis during different periods of S phase and how these might relate to the next-in-line model of S phase progression [28],[29]. As described above, such changes are presumably linked to changes in the local chromatin environment, which modulates the efficiency with which potential origins are established and used.

While the ability to relate replication timing to DNA sequence and chromatin features, such as histone modifications, is compelling [10]–[15], one limitation of studies based on cell populations is that any cell-to-cell variability is lost. This is inevitable as population-based approaches will smooth any biological complexity that we might expect to see as experimental noise. In contrast, analysis of DNA synthesis within individual nuclei and on isolated DNA fibers [5],[20], is able to reveal detail related to the specific events that occur within individual cells. However, despite obvious experimental differences, our attempt to integrate data from genome-wide and single cell studies has shown that replicon clusters within domains that contain ∼500 kbp of DNA provide the functional targets during replication of mammalian genomes (Figure 5). Moreover, evidence discussed above shows how data derived from single cells and cell populations support a general model for S phase progression that is in part based on the stochastic activation of potential replication origins and in part on the sequential activation of replication domains, based on their genetic continuity along chromosomes.

### A model of S phase progression

**Figure 7:**
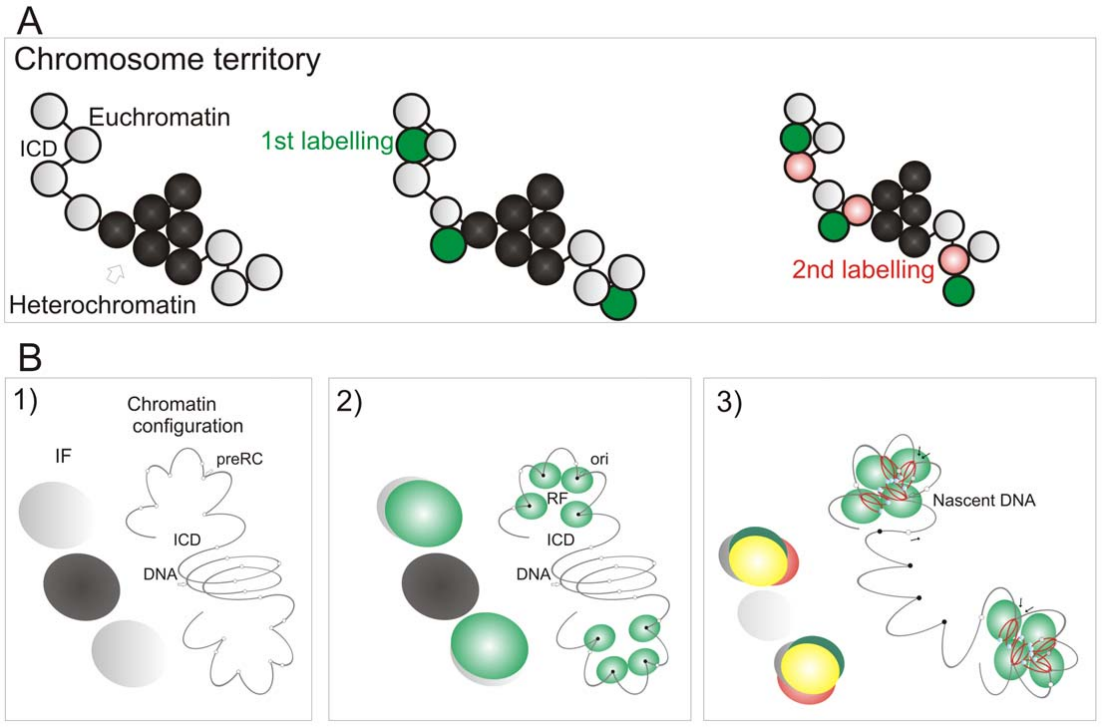
A model linking the organization of replicon clusters to S phase progression. A model (A,B) for S phase progression shows how the spatial and genetic continuity of DNA foci together with the organization of DNA foci and CTs relative to the interchromatin domain regulate the selection of active foci as S phase proceeds. CTs (A, one is shown) are composed of discrete DNA foci (coloured spheres), with structural characteristics that are defined by the epigenetic status of DNA to yield open and accessible euchromatic foci (grey) or more condensed and relatively inaccessible heterochromatic foci (black). The structure, accessibility—relative to the inter-chromatin domain (ICD)—and sequential labeling of adjacent foci provide 3 key determinants that define the course of S phase (B). Potential initiation sites (pre-RC complexes—small open circles) scattered throughout the chromatin fiber (line) interact by chance with the replication machinery (small green circles; B2) to initiate synthesis at a fraction of pre-RCs (now functional origins—small filled circles) within a local replication factory (RF—of clustered replisomes). As synthesis continues, chromatin fibers are reeled into the active synthetic factory and nascent strands displaced from the factory surface (B3). Eventually, the internal forks from adjacent replicons fuse and terminate. The outgrowing forks continue to grow and at some point structural changes in genetically linked chromatin (B3) increase the probability of activating origins within the adjacent foci. Three large spheres on the left of each panel in (B) depict the structures that would be visualized using fluorescent microscopy (IF): Grey—the structure of DNA foci that would be seen by prior labeling in vivo (for example with Cy3-dUTP); Green—location of active replication complexes and factories; Red—the nascent DNA; Yellow—overlap of red and green structures.

The preferential accessibility of potential origins within open chromatin and the differential sensitivity of early and late origins to different cyclin/CDK complexes are major regulators of origin selection. These properties then dictate the efficiency with which different loci – such as potential replication origins (pre-RCs; Figure 7B) – interact with the inter-chromatin compartment where active replication factories are formed (RF; Figure 7B2). Origin selection is never-the-less stochastic, as most potential origins are replicated passively throughout S phase [6]. However, once S phase has begun, our data suggest that a next-in-line principle [28],[29] defines the efficiency with which origins can be activated in the downstream replication program, so that only a minority (at most 10%) of *de novo* initiation events are uncoupled from synthesis within previously active replicon clusters (Figure 4). As replication within engaged replicon clusters approaches completion, the external forks might drive structural perturbations in neighboring foci that alter the exposure of potential origins to the replication machinery and so increases the probability of their activation (Figure 7B3). In this way, the genetic continuity of DNA foci along the chromosomal fiber provides a fundamental determinant of S phase progression in mammalian cells.

In the absence of genetically defined initiation sites, it is interesting to speculate how the mammalian cells have evolved to ensure that their genetic information is preserved during cell proliferation. Given the demand for precision, it is perhaps surprising that a key regulatory principle involves the random activation of potential initiation sites that are significantly more numerous than necessary to perform synthesis on schedule [33]. This stochastic feature of initiation and the redundancy of potential origins ensures that the system has sufficient tolerance to complete synthesis on schedule if the synthetic environment happens to change; any condition that result in slowing or stalling of the engaged forks are counteracted by local increases in origin density [reviewed in 42]. This regulatory mechanism operates at the level of DNA foci, and recent studies have suggested that a replication-dependent memory mechanism, based on the structure of DNA loops, ensures that appropriate levels of synthesis are maintained from one cell cycle to the next [43].

During S phase, the co-ordinated activation of groups of replicons within DNA foci will reduce the number of active synthetic sites that are required to complete synthesis. In addition, as replicon clusters engage synthesis together, within dedicated replication factories [18], this organization minimizes the time that adjacent replicons are replicating before their growing forks meet and fuse to terminate synthesis. Growing forks are complex structures that are inevitably more prone to damage and recombination than DNA packaged into normal chromatin, hence limiting the number of exposed forks will minimize the risk of damaging the genome. In addition, the sequential activation of replicon clusters based on their genetic continuity along chromosomes will also limit the number of isolated forks. Hence, we propose that the orderly synthesis of replicon clusters within DNA foci has evolved as a mechanism to ensure that higher eukaryotes can duplicate their genomes with the required efficiency while ensuring the preservation of both genetic and epigenetic information.

## Materials and Methods

### Labeling replication foci in situ

HeLa cells were grown in DMEM (Sigma) with 5% FBS and antibiotics. MRC5 cells were grown in MEM with 10% FBS and antibiotics. Replication foci were pulse-labeled in culture medium containing 10 µM bromo-deoxyuridine (BrdU) or labeled with modified replication precursor analogues: Cy3-; AlexaFluor488- (AF488-); biotin-; or digoxigenin-dUTP as described by Maya-Mendoza et al. [44]. Active replication factories were defined by transient expression of GFP-PCNA [29] or indirect immuno-fluorescence with a PCNA specific antibody (Immuno Concepts; Auto I.D. serum No 6006; 1/1000; 15 h; 4°C). Chromatin was visualized by transient expression of DsRed-histone-H2B. Unsynchronized cells were used throughout this study; this was a deliberate choice to avoid synchrony-dependent artefacts and preserve the natural structure of the S phase program.

### DNA fiber experiments

DNA fiber spreads were prepared as previously described [19],[44] using very low densities of labeled cells – of 10^3^ cell/spread only 5–10 were labeled in these experiments. This low density minimizes DNA bundles and tangles within labeled fibers and allows visualization of Mbp fibers. In addition, the low density of labeled cells allows analysis of fibers from individual labeled cells. BrdU labeled tracks were detected with BrdU anti-sheep antibody (Biodesign; M20105S; 1∶1000 dilution; 1 h at 20°C) and biotin-11-dUTP tracks using a mouse monoclonal antibody (Clone BN-34, Sigma; 1∶1000 dilution; 1 h; 20°C). Primary antibodies were detected using Cy3- or AF488-conjugated donkey anti-sheep and anti-mouse secondary antibodies. The slides with DNA fibers were mounted with 50∶50 PBS-glycerol.

Fibers were examined using a Zeiss LSM 510META confocal microscope using a 40× lens, labeled tracks measured using the LSM software and converted to kbp using a conversion factor of 1 µm = 2.59 kbp [19]; under these imaging conditions a single imaging field contains ∼0.8 Mbp DNA. Double-labeled fibers were imaged only in dispersed, untangled areas of the DNA spread, to ensure the continuity of adjacent replicon clusters on individual DNA fibers. Routine quality control for spreading was performed using direct DNA labeling with YOYO-1 (Figure S9F) or cells labeled for >24 h with 10 µM BrdU, to give fully Br-labeled fibers (Figure S9G).

### Immuno-fluorescence and direct labeling of DNA foci

DNA foci labeled with BrdU, biotin-dUTP or digoxigenin-dUTP were visualized by indirect immuno-fluorescence as described [19],[44]. Cells were grown on coverslips, pulse labeled (directly or by transfection) and fixed in 4% paraformaldehyde. Fixed cells were acid treated (for BrdU labeling) and washed 3× in PBS, treated with 0.5 Triton ×100 in PBS, rinsed 3× in PBS, 3× PBS+ (PBS plus 1% BSA and 0.1% Tween 20), blocked for 1 h and incubated for 1 h with the appropriate antibody. Secondary antibodies were conjugated with Cy3, AF488, AF647 and Qdot reagents (Invitrogen). For 2^nd^ or 3^rd^ pulse detection, cells incubated after first detection including secondary antibody, were washed 3× in PBS and 3× in PBS+ and incubated with the appropriate first and second antibodies. In some experiments we used BrdU anti-rat (Immunologicals Direct Clone BU 1/75; 1∶1000 dilution; 1 h; 20°C) and a secondary anti-rat antibody conjugated with Qdot-605. Streptavidin-Qdot-525 was used to identify sites containing biotin-dUTP. Finally, slides were washed 3× in PBS+, 3× in PBS, incubated with 5 µg/ml Hoechst 33258 (Sigma) for 10 min, rinsed 3× in PBS and mounted with either Vectashield or Prolong mounting media. Mitotic chromosomes were prepared as described [44].

For confocal imaging, samples were examined using a Zeiss LSM 510META confocal microscope and 100× (1.45 NA) lens. 3D images were generated using Z stacks and processed in Imaris software. In order to ensure optimal imaging performance, instrument alignment was performed at regular intervals by Zeiss. Chromatic shift was corrected using multi-coloured TetraSpeck florescent beads; the maximum tolerated shifts were 50 nm in X–Y and 100 nm in Z (Figure S3B). To minimize chromatic shift, for all experimental conditions extreme care was taken to balance labeling intensities in different imaging channels. In addition, for each indirect labeling experiment multiple samples were prepared so that each replication pulse could be labeled with the different secondary reagents used. 4D time-lapse imaging was performed using a Deltavision microscope with a CoolSNAP-HQ2 camera and Olympus objective (100x; 1.4 NA). The intensity of light during imaging was kept to 32% using an acquisition speed of 100–200 ms. Chromosome spreads were captured using a Deltavision microscope and images deconvolved using 5–10 iterations and pre-filter cut-off values (microns) of 0.05.

The 3D and 4D images were analyzed using Imaris software. For LSM images of individual CTs a 0.02 µm Gaussian filter was applied. For nearest neighbor analysis, 3D projections were generated in Imaris software from confocal Z series and software used to identify 3D labeled sites and the mass centers of individual sites (foci). Individual channels were processed separately. The co-ordinates of the mass centers were then used to define the spatial relationship between adjacent foci, either within or between channels. For presentation, the imaging software represents the mass centers of DNA foci as computer generated spheres that correspond in size to average foci. Images generated in doing this are clearly artificial and while providing an accurate representation of the positions of foci are not intended to provide a realistic representation of the foci themselves.

### Bioinformatic analysis of replication timing domains

Replication timing data from human ES cells [10] was taken from the Integrative Genomics Viewer website at: http://www.broadinstitute.org/igv. For analysis, we choose to use human chromosome 6, as we have used this chromosome recently to model S phase [45]. To map the replication timing domains, ∼10 Mbp regions were selected at random and points of inflection defined to identify peaks in the timing profile. Distances between adjacent peaks were then taken from the browser to develop a profile of distributions.

Profiles of distributions for replication foci were generated using parameters for the distribution of replicons per cluster and the length of replicons [19]. For simulation, the primary data for replicon length was approximated to a normal distribution (μ = 140.6238kbp, σ = 58.8192), which was then sampled to determine the length of each individual replicon and assimilated into replicon clusters using the published frequencies of replicons/cluster. Simulations were implemented in Matlab.

## Supporting Information

Figure S1 Three colour labeling to assess the spatial continuity of replication foci at different times of S phase. Nascent DNA synthesis in unsynchronized HeLa cells was labeled by indirect immuno-fluorescence after consecutive incorporation pulses using combinations of biotin-dUTP (blue), digoxigenin-dUTP (green) and BrdU (red). In some experiments the active factories were labeled using antibodies to PCNA (red). High-resolution 3D confocal images (1 µm sections are shown) of typical examples demonstrate how the 3 channel labeling can be utilized to define the structure of individual sites and the spatial continuity that links the separate pulses. Mid/late S phase patterns (A,C) provide discrete foci with clear structure and spatial connectivity. In early S phase, in contrast (B), while differentially labeled domains within individual foci can be identified with ease the complexity of the foci means that foci labeled during consecutive time zones of S phase will inevitable lie in close proximity. For (A–C), boxed areas in panel 1 are shown at high magnification in panels 2 and 3 and the intensity plots in panel 4 are scans along the line indicated in panel 2. The labeling protocol is shown on the left of the figure. Because cells were fixed immediately after incorporation, any labeling asymmetry presumably reflects the synthetic polarity that arises when DNA foci are replicated by a dedicated synthetic factory. Scale bars: 5 and 0.5 µm.
(9.44 MB TIF)

Figure S2 Spatio-temporal relationship of active replication factories and DNA foci. To establish the temporal separation between replication foci labeled during different replication time zones (A) HeLa cells were pulse labeled with biotin-dUTP (red), chased for 30, 60, and 120 min in medium and pulse labeled with BrdU (green). Separation of individual foci was seen following an intervening chase period of ∼60 min in early S phase and ∼120 min during mid and late S phase (A and insets at high magnification). (B) shows the percentage of imaging voxels in which the two precursors co-localized during early S phase following different chase intervals using 3D imaging (n = 25 nuclei/sample). (C) shows the size of replication foci during early, mid and late S phase (n = 200 for each pattern). Scale bars: 5 and 0.5 µm.
(9.22 MB TIF)

Figure S3 Chromatic shift influences the precision of co-localization during spatial analysis of DNA foci. HeLa cells were transfected at the same time using 488-dUTP and Cy3-dUTP, cultured for 7 days and chromatic shift evaluated (A). Confocal sections of individual imaging channels were recorded and mass centers (maximal intensities) of labeled foci defined by Imaris imaging software. Distances between the identified centers of labeled sites were then measured (78.37+/−53.48 nm shift, n = 68) to define the extent of chromatic shift. Chromatic shift due to instrument alignment was corrected using multi-coloured TetraSpeck florescent beads (B) — the maximum tolerated shifts were 50 nm in X–Y and 100 nm in Z; alignment was performed at regular intervals by Zeiss engineers. Scale bars: 1 and 2 µm in (A) and (B), respectively.
(6.78 MB TIF)

Figure S4 Structural analysis of DNA foci in individual CTs. Replication foci of unsynchronized HeLa cells were pulse-labeled to incorporate selected replication precursor analogues into nascent DNA. Cells were labeled with consecutive pulses of biotin-dUTP and BrdU both without (A) and with (B) an intervening 1h chase. Cells were then grown for 6–7 days to resolve the labeled CTs. After this time, cells with discrete labeled territories were analyzed using confocal microscopy. Pseudo-shapes were generated by image processing software to define the boundaries of labeled foci. In this example, shapes defined by the biotin labeling are transposed onto the other images to demonstrate the separation of labels in the different channels. In some experiments, CTs were also labeled with Qdot-conjugated secondary antibodies (C) to allow increased section density and Z resolution. (D) shows single channel (eg biotin to biotin or BrdU to BrdU) nearest neighbor analyzes for the labeled DNA foci within individual CTs. Scale bars: 5 and 0.5 µm.
(7.39 MB TIF)

Figure S5 Chromosome territories in human fibroblasts. CTs of MRC5 cells were analyzed after 6–7 days in culture. Cells were pulse labeled with biotin-dUTP (30 min; red) and subsequently with BrdU (20 min; green) following growth in fresh medium for 0, 1, or 2 h. Cells were fixed and sites of incorporation detected using indirect immuno-fluorescence and confocal microscopy; projections of confocal Z-stacks are shown. Using the pulse-chase (1 h)-pulse strategy, labeled early S phase foci of MRC5 cells were 513+/−116 nm (n = 200) in diameter and foci labeled during the 1^st^ and 2^nd^ pulses were 556+/−114 nm (n = 155) apart. Scale bars: 5 µm.
(9.12 MB TIF)

Figure S6 Different models of S phase progression. During S phase, the distribution of active sites that is defined by incorporation of labeled nucleotides into DNA foci allows identification of early, mid and late S phase cells. Multiple pulses with different timing separations can be used to monitor transitions between these different periods (A). However, DNA foci within the nuclear space are so highly crowded that defining the molecular principles that underlie the timing program is technically challenging. Three obvious models might account for the structure of the timing program. (A,1) - the genetic continuity between foci might provide an innate mechanism that allows foci to be replicated in a particular pattern once a specific set of foci is activated at the onset of S phase. (A,2) – a mechanism of spatial continuity might operate if once active factories are assembled the subsequent completion of synthesis allows factories to interact with the nearest unreplicated DNA foci. If factories disassemble when synthesis is complete, decay of active sites might provide a local high concentration of synthetic components that stimulates the assembly of new factories within the same nuclear domain. (A,3) – random activation of DNA foci within distinct chromatin compartments – eg euchromatin and heterochromatin – might explain the timing program if, for example, different CDK/cyclin complexes are required to activate origins within different chromatin compartments. (A) shows how these different models can be analyzed using the distribution of labeled foci within individual CTs during interphase and single chromosomes during metaphase. Random S phase progression can be modeled using statistical tools and MathLab software (B). Two examples are shown (B), which mimic the appearance of confocal sections. To simulate foci within diploid mammalian nuclei we generated random distributions of 350 spheres with 500 nm diameter – the foci - within a single large sphere of 10 µm diameter – the nucleus (Figure S6B). We assumed that S phase contained 10 time zones of 1 hour each so that 10% of foci were active at any particular time. With these assumptions, nuclei contain a total of 3500 foci that would occupy 44% of the total nuclear volume, as expected in proliferating diploid mammalian cells. Notably, the randomly generated patterns displayed similar structural features to foci seen during early S phase, yet when two randomly generated channels (single colour images) were overlaid (double colour images) the 1∶1 co-association of nearest red and green neighbors that was seen experimentally in cells was never reproduced. The importance of spatial continuity is clearly evident in labeled cells, even immediately following labeling when the density of labeled foci is too high to allow detailed analysis in early S phase (C), though analysis in mid S phase (D) is possible. The same conclusion is reached if labeled cells are grown prior to analysis to resolve the labeled CTs by random chromosome segregation (E,F). Using precursors that can be imaged without processing, during both interphase (E) and metaphase (F), chromosomes labeled using a pulse-chase (2h)-pulse strategy always retain a high degree of co-association between sites labeled with the 1^st^ and 2^nd^ pulse labels. Using this live cell imaging approach, all CTs analyzed during interphase correspond with individual labeled chromosomes during metaphase. Scale bars: 5 and 0.5 µm in (C,D), and 10 µm in (E,F).
(8.74 MB TIF)

Figure S7 Three colour labeling to assess the genetic continuity of replication foci in chromosome territories. HeLa cells were labeled with sequential pulses of AF488-dUTP, Cy3-dUTP and BrdU each separated by unlabeled periods of 1 h (A). After 7 days, cells were fixed and BrdU detected using indirect immuno-labeling with rat anti-BrdU and anti-rat IgG conjugated with AF647 (B). Individual image channels were recorded for each precursor and the mass centers for individual foci defined by Imaris imaging software. Nearest neighbor analysis was then performed using all possible pair-wise combination (C): 1^st^–2^nd^ pulses = 414.88+/−111.36 nm; 2^nd^–3^rd^ = 376.96+/−109.64 nm; 1^st^–3^rd^ = 487.17+/−137.66 nm; n = 150. Scale bars: 5 and 1 µm, as indicated on individual panels.
(9.67 MB TIF)

Figure S8 Extended pulse separations preclude nearest neighbor analysis. HeLa cells were pulse-labeled with AF488-dUTP, chased for 4 or 5 h and pulse-labeled with Cy3-dUTP. After 7 days, cells were fixed and images collected. As before, individual CTs contain distinct labeled sites of ∼400 nm, which correspond to DNA foci that are labeled with the different precursors. Under these conditions, all sites are labeled uniquely with only one precursor. Moreover, patterns of foci labeled in the two channels are clearly unrelated, with foci labeled during the 1^st^ and 2^nd^ pulses populating distinct regions of individual CTs. CTs within 2 typical cells are shown. The magnified image (below) is a 2.5× view of the region highlighted (boxed area, above). Separate imaging channels and a channel merge are shown. Scale bars: 10 and 5 µm, as indicated on individual panels.
(6.65 MB TIF)

Figure S9 Structure analysis of DNA fibers defines genetic continuity during the S phase progression. HeLa cells were pulse-labeled (30 min) with biotin-dUTP grown for 1 h in medium and then pulse-labeled (20 min) with BrdU. DNA fibers from the labeled cells were spread on to glass slides and active replicons visualized by confocal microscopy after indirect immuno-labeling. Double labeled fibers of ∼1–2 Mbp in length were recorded and analyzed. Typical examples of stalled replication forks (A) and long extending replicons (B) are shown. The analysis of the distance between replication forks (C; distances measurements using Zeiss software are superimposed on the images) correlates well with the labeling and chase times used, given rates of synthesis in the range 1–2 kb/min/fork. Using 5–10 cells/spread, almost all biotin-labeled fibers contain associated forks that are labeled with BrdU (see typical examples shown in C). A minority – 5% in each of 4 experiments (144 image fields like those shown) – of fibers in the double labeled regions of a spread were labeled only with BrdU (D shows typical image fields; n = 144). This suggests that de novo initiation events that occur as S phase proceeds are almost always coupled to existing active sites. The average separation of origins in clusters with extending forks and *de novo* (secondary) activation of adjacent clusters was 181.2+/−87.5 kbp and 119.6+/−47.0 kbp, respectively (E). DNA fiber integrity and distribution was assessed routinely by YOYO-1 staining—typical staining of a biotin-labeled sample is shown (F). DNA fiber integrity during BrdU labeling is also evident from the integrity of the labeled fibers—staining of biotin labeled forks on a fully labeled DNA fibre are shown (G). *In situ* labeling, using the same labeling program (H), shows how the complex patterns of incorporation into replication foci (foci 1–3) can be attributed to the distribution of replication structures on nascent DNA fibers (replicons shown in cartoon form below). Scale bars: 50 µm in (D), 5 and 0.5 µm in (F).
(9.18 MB TIF)

Figure S10 Using genome-wide and single cell approaches to analyze replication timing. (A–C) show the structure of 3 well-characterised examples of initiation sites for mammalian DNA synthesis. At some sites, local gene structure determines that replication might initiate at a specific site (A)—the human lamin B2 locus represents a paradigm for this class of origin. Some replicons have dispersed potential sites of initiation, which contain preferred initiation sites within them (B)—the mammalian DHFR locus is a good example of this class of initiation domain. Finally, some loci contain regions (C) with hotspots of replication initiation that contain many possible sites within clusters of potential origins that cover about 10 kbp. The example shown contains 4 potential initiation zones, which may be treated as individual replicons (C1–4), but in the cells can be activated unpredictably—selection is stochastic—so that different cells initiate synthesis from different sites across the locus [see 20 for details]. The cartoon in (D) depicts an imaginary DNA locus of ∼1 Mbp, which contains each of these three classes of initiation domain. In the cell, this locus would fold to occupy a single DNA focus. Analysis of replication across the locus using DNA fibres isolated from individual cells would reveal a range of patterns, such as the two depicted in (D1–2). However, a genome-wide analysis designed to define replication timing across the locus (D3) would give a more complex picture that incorporates all possible initiation events across the cell population used.
(0.42 MB TIF)

Video S1 Time-lapse analysis of DNA foci dynamics—consecutive pulse labels. The time-lapse series from the experiment in Figure 2B shows how individual foci labeled with consecutive pulses are dynamic within CTs so that adjacent sites labeled with the 1^st^ and 2^nd^ precursor always maintain complete co-association. Using a live cell imaging protocol that maintains cell viability for at least 24 h, images shown were taken at 15 min intervals for 3 h. Video S1 shows the mobility of foci directly (1 frame/second), without further processing.
(0.69 MB MOV)

Video S2 Time-lapse analysis of DNA foci dynamics—consecutive pulse labels. A representation of Video S1 in which image processing software was used to replace each labeled site in the green (1^st^) and red (2^nd^) channels with a sphere of 500 nm; the spheres and original labeled sites have coincident centers of mass. Individual images in the video are presented at a rate of 1 frame/second.
(0.34 MB MOV)

Video S3 Time-lapse analysis of foci dynamics—consecutive pulse labels with an intervening 1 h unlabeled period. The time-lapse series from the experiment in Figure 2C was prepared as described in the legend to Video S1. Even with 1 h and 2 h (not shown) unlabeled periods between the two pulses, foci containing the 1^st^ and 2^nd^ precursors maintain complete spatial co-association over an imaging time course of 3 h. As CT shape changes significantly over the imaging time course, the persistent co-association of neighboring foci is clearly consistent with them being genetically linked along chromosomes.
(0.18 MB MOV)
