# Supplementary figures and images for: S Phase Progression in Human Cells Is Dictated by the Genetic Continuity of DNA Foci
Source: PLoS Genet. 2010 Apr 8;6(4):e1000900. doi: 10.1371/journal.pgen.1000900 (PMC2851568; doi:10.1371/journal.pgen.1000900)

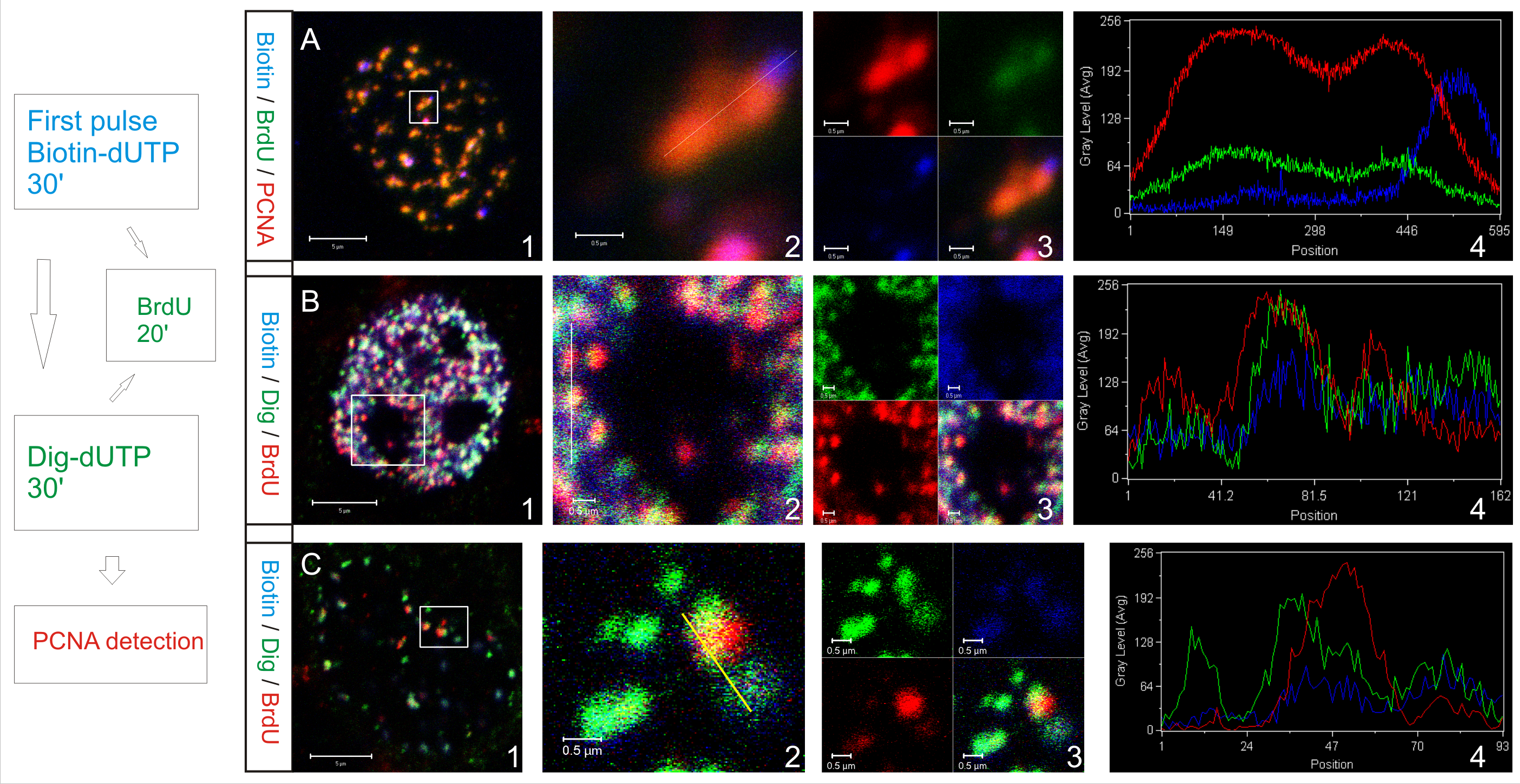

Supplement: Figure S1 — Three colour labeling to assess the spatial continuity of replication foci at different times of S phase. Nascent DNA synthesis in unsynchronized HeLa cells was labeled by indirect immuno-fluorescence after consecutive incorporation pulses using combinations of biotin-dUTP (blue), digoxigenin-dUTP (green) and BrdU (red). In some experiments the active factories were labeled using antibodies to PCNA (red). High-resolution 3D confocal images (1 µm sections are shown) of typical examples demonstrate how the 3 channel labeling can be utilized to define the structure of individual sites and the spatial continuity that links the separate pulses. Mid/late S phase patterns (A,C) provide discrete foci with clear structure and spatial connectivity. In early S phase, in contrast (B), while differentially labeled domains within individual foci can be identified with ease the complexity of the foci means that foci labeled during consecutive time zones of S phase will inevitable lie in close proximity. For (A–C), boxed areas in panel 1 are shown at high magnification in panels 2 and 3 and the intensity plots in panel 4 are scans along the line indicated in panel 2. The labeling protocol is shown on the left of the figure. Because cells were fixed immediately after incorporation, any labeling asymmetry presumably reflects the synthetic polarity that arises when DNA foci are replicated by a dedicated synthetic factory. Scale bars: 5 and 0.5 µm. (9.44 MB TIF) [file pgen.1000900.s001.tif]

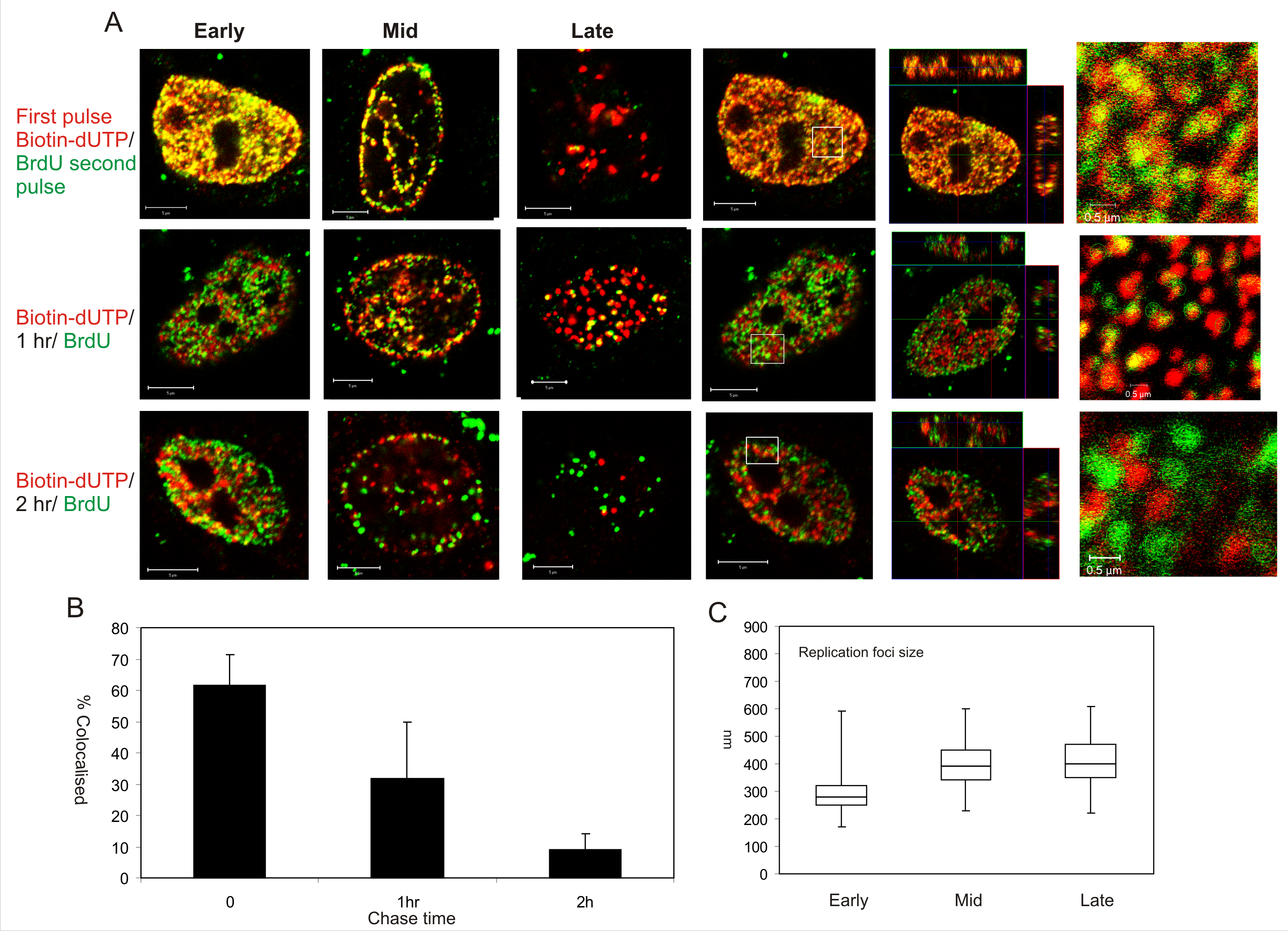

Supplement: Figure S2 — Spatio-temporal relationship of active replication factories and DNA foci. To establish the temporal separation between replication foci labeled during different replication time zones (A) HeLa cells were pulse labeled with biotin-dUTP (red), chased for 30, 60, and 120 min in medium and pulse labeled with BrdU (green). Separation of individual foci was seen following an intervening chase period of ∼60 min in early S phase and ∼120 min during mid and late S phase (A and insets at high magnification). (B) shows the percentage of imaging voxels in which the two precursors co-localized during early S phase following different chase intervals using 3D imaging (n = 25 nuclei/sample). (C) shows the size of replication foci during early, mid and late S phase (n = 200 for each pattern). Scale bars: 5 and 0.5 µm. (9.22 MB TIF) [file pgen.1000900.s002.tif]

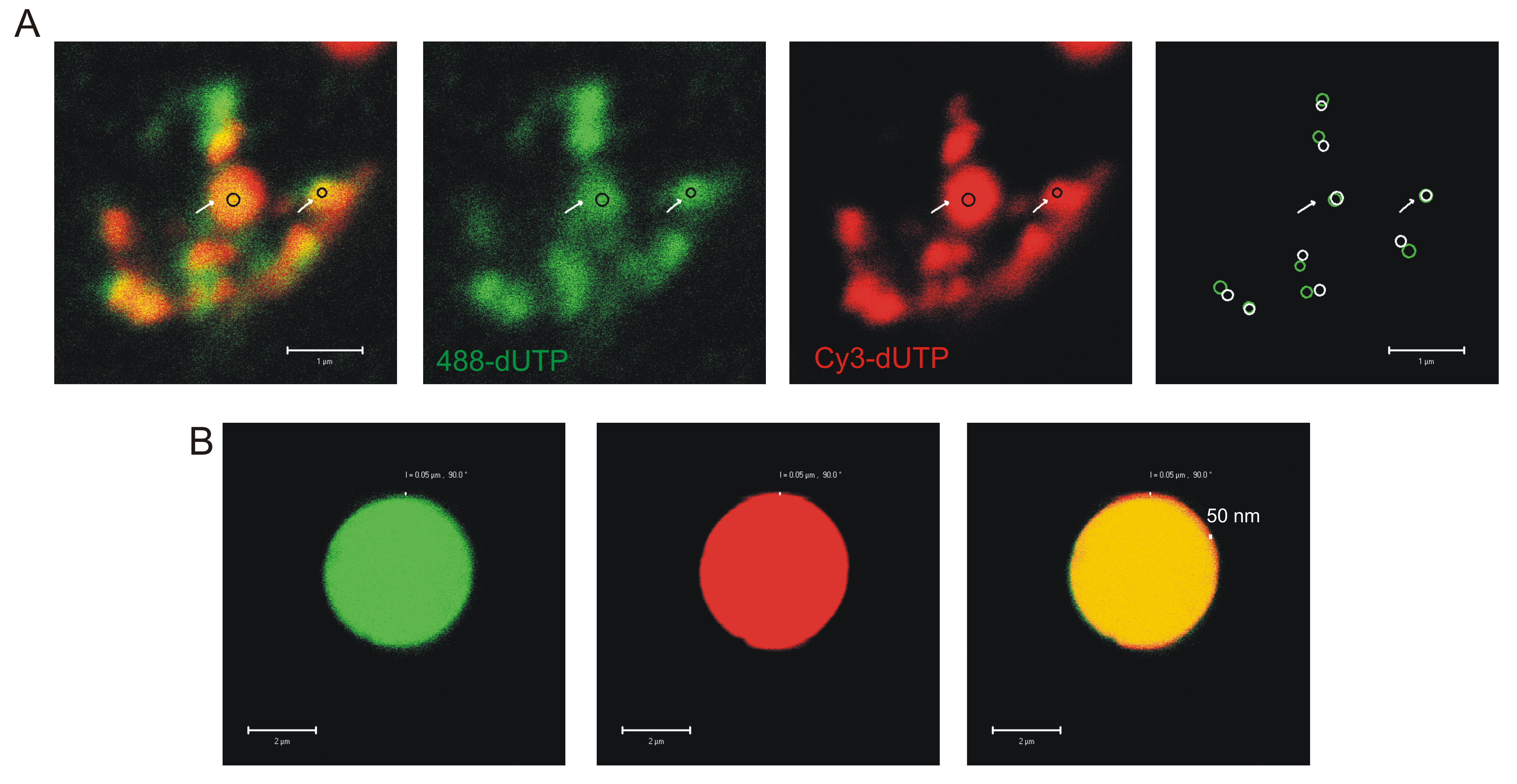

Supplement: Figure S3 — Chromatic shift influences the precision of co-localization during spatial analysis of DNA foci. HeLa cells were transfected at the same time using 488-dUTP and Cy3-dUTP, cultured for 7 days and chromatic shift evaluated (A). Confocal sections of individual imaging channels were recorded and mass centers (maximal intensities) of labeled foci defined by Imaris imaging software. Distances between the identified centers of labeled sites were then measured (78.37+/−53.48 nm shift, n = 68) to define the extent of chromatic shift. Chromatic shift due to instrument alignment was corrected using multi-coloured TetraSpeck florescent beads (B) — the maximum tolerated shifts were 50 nm in X–Y and 100 nm in Z; alignment was performed at regular intervals by Zeiss engineers. Scale bars: 1 and 2 µm in (A) and (B), respectively. (6.78 MB TIF) [file pgen.1000900.s003.tif]

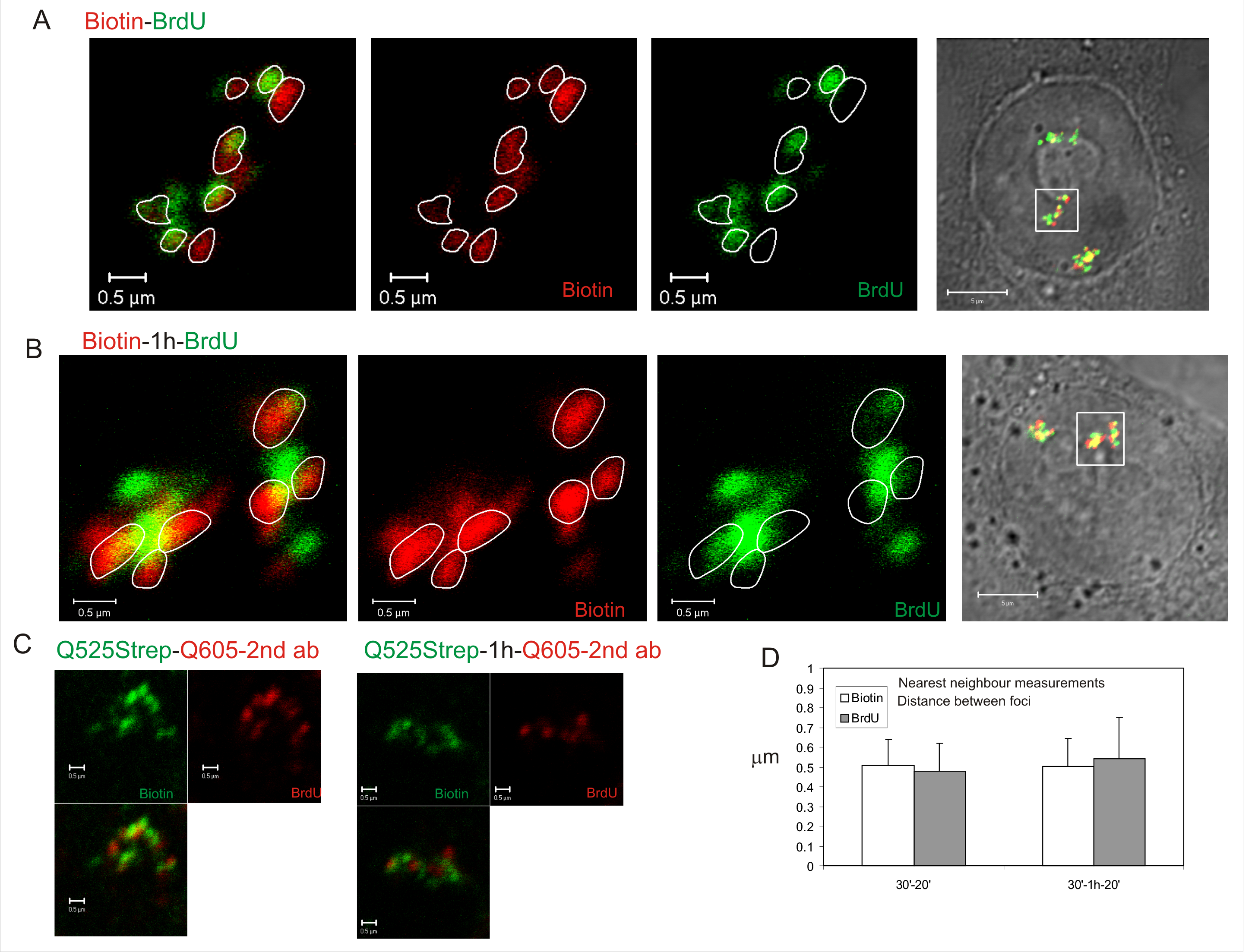

Supplement: Figure S4 — Structural analysis of DNA foci in individual CTs. Replication foci of unsynchronized HeLa cells were pulse-labeled to incorporate selected replication precursor analogues into nascent DNA. Cells were labeled with consecutive pulses of biotin-dUTP and BrdU both without (A) and with (B) an intervening 1h chase. Cells were then grown for 6–7 days to resolve the labeled CTs. After this time, cells with discrete labeled territories were analyzed using confocal microscopy. Pseudo-shapes were generated by image processing software to define the boundaries of labeled foci. In this example, shapes defined by the biotin labeling are transposed onto the other images to demonstrate the separation of labels in the different channels. In some experiments, CTs were also labeled with Qdot-conjugated secondary antibodies (C) to allow increased section density and Z resolution. (D) shows single channel (eg biotin to biotin or BrdU to BrdU) nearest neighbor analyzes for the labeled DNA foci within individual CTs. Scale bars: 5 and 0.5 µm. (7.39 MB TIF) [file pgen.1000900.s004.tif]

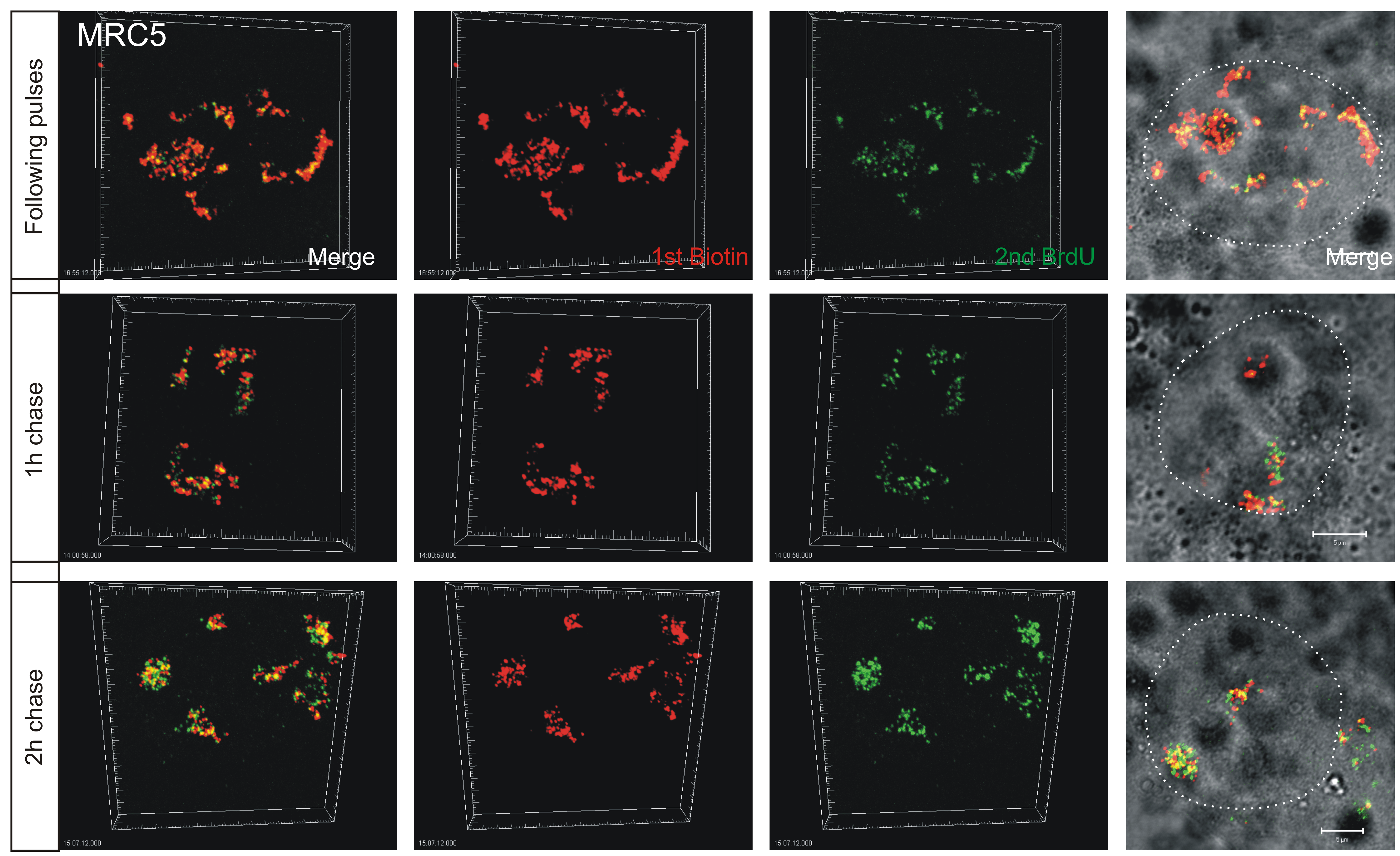

Supplement: Figure S5 — Chromosome territories in human fibroblasts. CTs of MRC5 cells were analyzed after 6–7 days in culture. Cells were pulse labeled with biotin-dUTP (30 min; red) and subsequently with BrdU (20 min; green) following growth in fresh medium for 0, 1, or 2 h. Cells were fixed and sites of incorporation detected using indirect immuno-fluorescence and confocal microscopy; projections of confocal Z-stacks are shown. Using the pulse-chase (1 h)-pulse strategy, labeled early S phase foci of MRC5 cells were 513+/−116 nm (n = 200) in diameter and foci labeled during the 1st and 2nd pulses were 556+/−114 nm (n = 155) apart. Scale bars: 5 µm. (9.12 MB TIF) [file pgen.1000900.s005.tif]

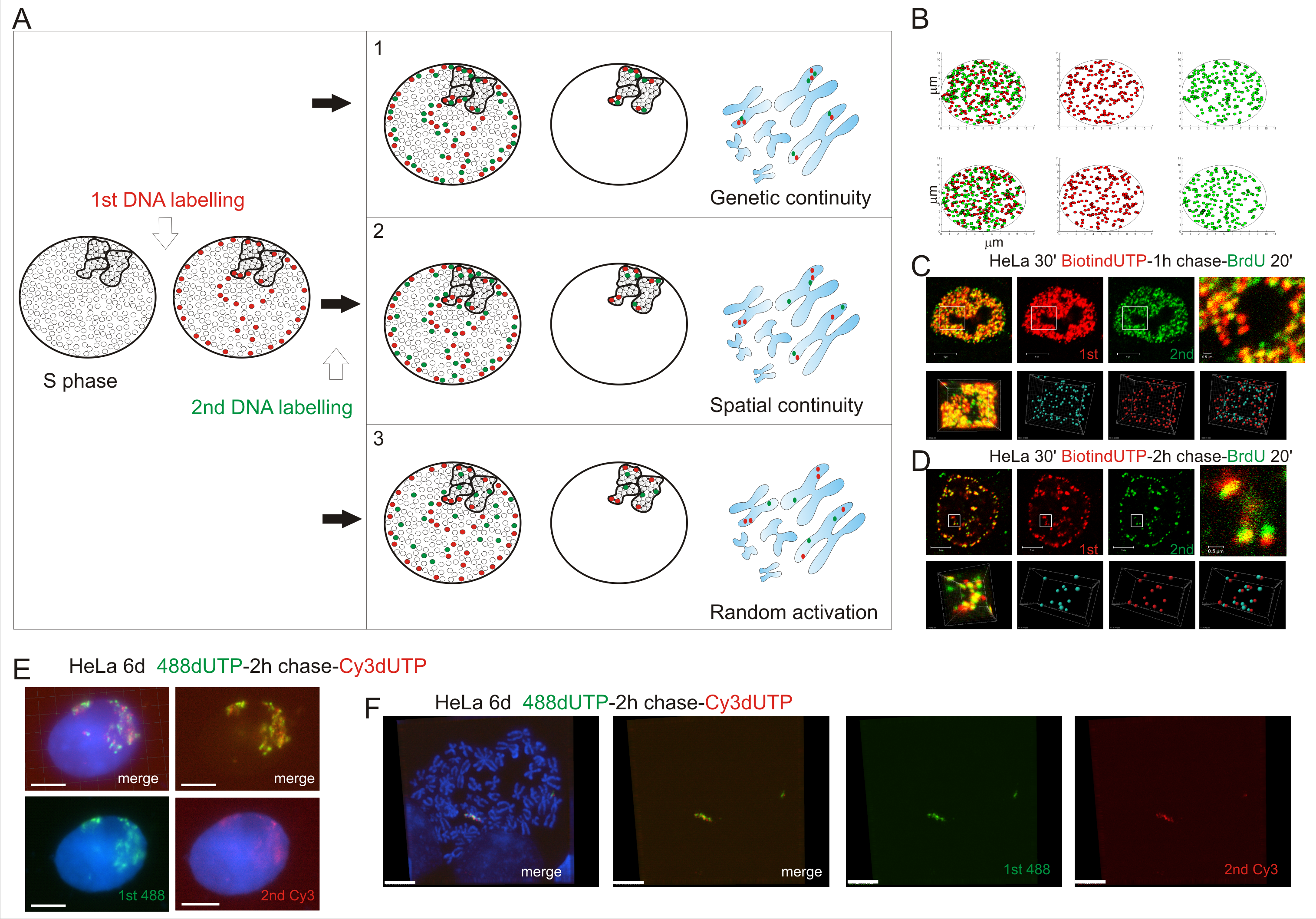

Supplement: Figure S6 — Different models of S phase progression. During S phase, the distribution of active sites that is defined by incorporation of labeled nucleotides into DNA foci allows identification of early, mid and late S phase cells. Multiple pulses with different timing separations can be used to monitor transitions between these different periods (A). However, DNA foci within the nuclear space are so highly crowded that defining the molecular principles that underlie the timing program is technically challenging. Three obvious models might account for the structure of the timing program. (A,1) - the genetic continuity between foci might provide an innate mechanism that allows foci to be replicated in a particular pattern once a specific set of foci is activated at the onset of S phase. (A,2) – a mechanism of spatial continuity might operate if once active factories are assembled the subsequent completion of synthesis allows factories to interact with the nearest unreplicated DNA foci. If factories disassemble when synthesis is complete, decay of active sites might provide a local high concentration of synthetic components that stimulates the assembly of new factories within the same nuclear domain. (A,3) – random activation of DNA foci within distinct chromatin compartments – eg euchromatin and heterochromatin – might explain the timing program if, for example, different CDK/cyclin complexes are required to activate origins within different chromatin compartments. (A) shows how these different models can be analyzed using the distribution of labeled foci within individual CTs during interphase and single chromosomes during metaphase. Random S phase progression can be modeled using statistical tools and MathLab software (B). Two examples are shown (B), which mimic the appearance of confocal sections. To simulate foci within diploid mammalian nuclei we generated random distributions of 350 spheres with 500 nm diameter – the foci - within a single large sphere of 10 µm diameter – [file pgen.1000900.s006.tif]

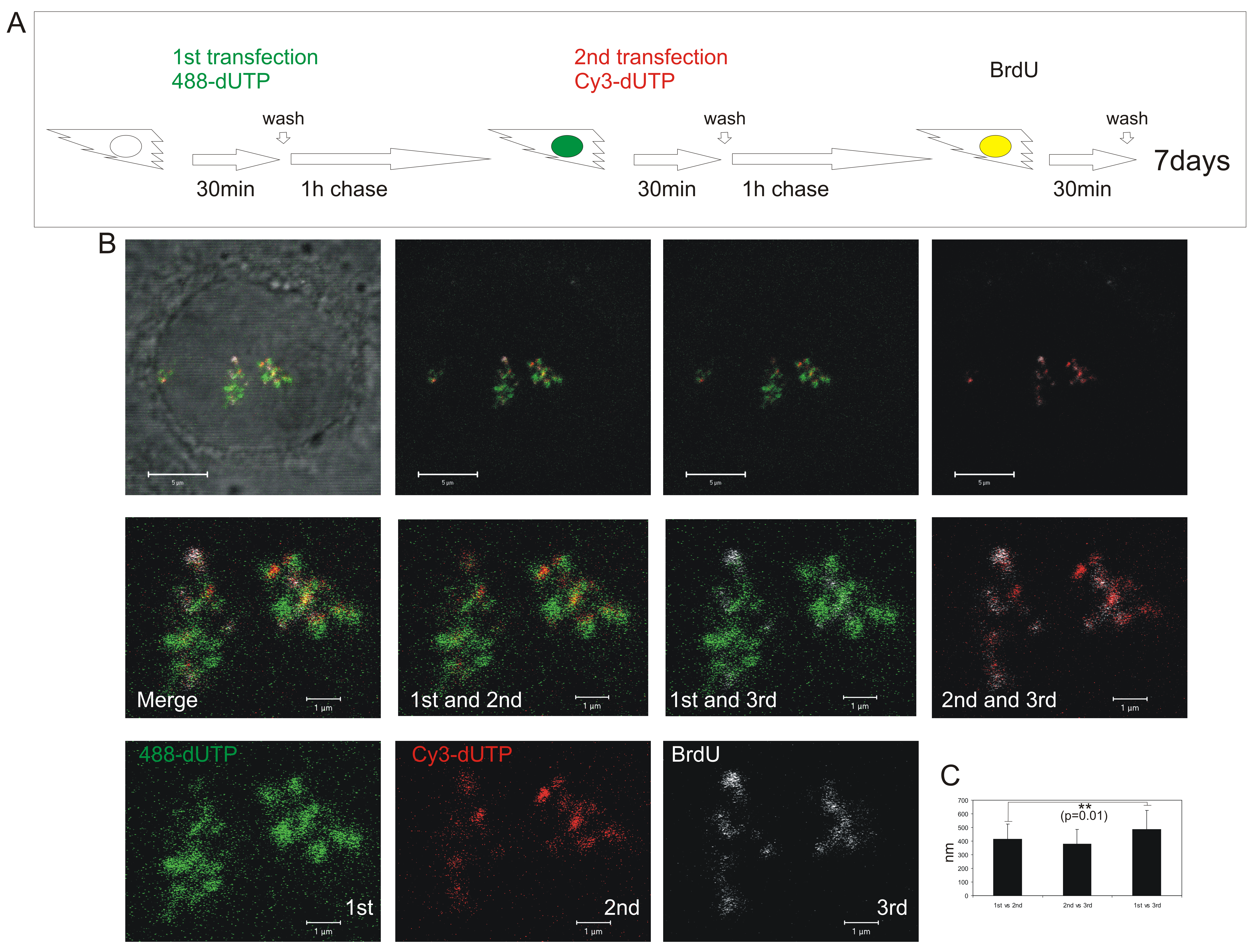

Supplement: Figure S7 — Three colour labeling to assess the genetic continuity of replication foci in chromosome territories. HeLa cells were labeled with sequential pulses of AF488-dUTP, Cy3-dUTP and BrdU each separated by unlabeled periods of 1 h (A). After 7 days, cells were fixed and BrdU detected using indirect immuno-labeling with rat anti-BrdU and anti-rat IgG conjugated with AF647 (B). Individual image channels were recorded for each precursor and the mass centers for individual foci defined by Imaris imaging software. Nearest neighbor analysis was then performed using all possible pair-wise combination (C): 1st–2nd pulses = 414.88+/−111.36 nm; 2nd–3rd = 376.96+/−109.64 nm; 1st–3rd = 487.17+/−137.66 nm; n = 150. Scale bars: 5 and 1 µm, as indicated on individual panels. (9.67 MB TIF) [file pgen.1000900.s007.tif]

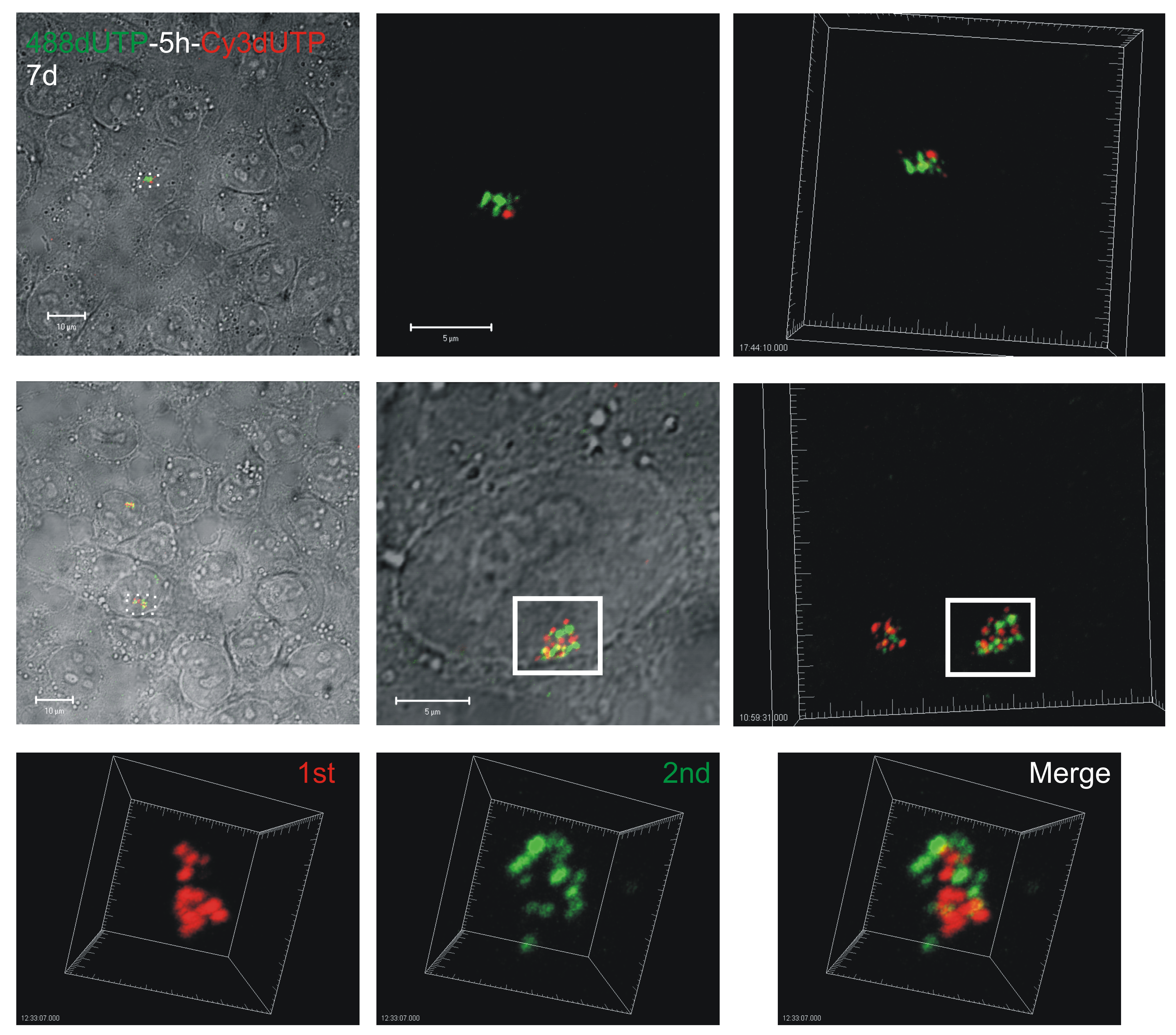

Supplement: Figure S8 — Extended pulse separations preclude nearest neighbor analysis. HeLa cells were pulse-labeled with AF488-dUTP, chased for 4 or 5 h and pulse-labeled with Cy3-dUTP. After 7 days, cells were fixed and images collected. As before, individual CTs contain distinct labeled sites of ∼400 nm, which correspond to DNA foci that are labeled with the different precursors. Under these conditions, all sites are labeled uniquely with only one precursor. Moreover, patterns of foci labeled in the two channels are clearly unrelated, with foci labeled during the 1st and 2nd pulses populating distinct regions of individual CTs. CTs within 2 typical cells are shown. The magnified image (below) is a 2.5× view of the region highlighted (boxed area, above). Separate imaging channels and a channel merge are shown. Scale bars: 10 and 5 µm, as indicated on individual panels. (6.65 MB TIF) [file pgen.1000900.s008.tif]

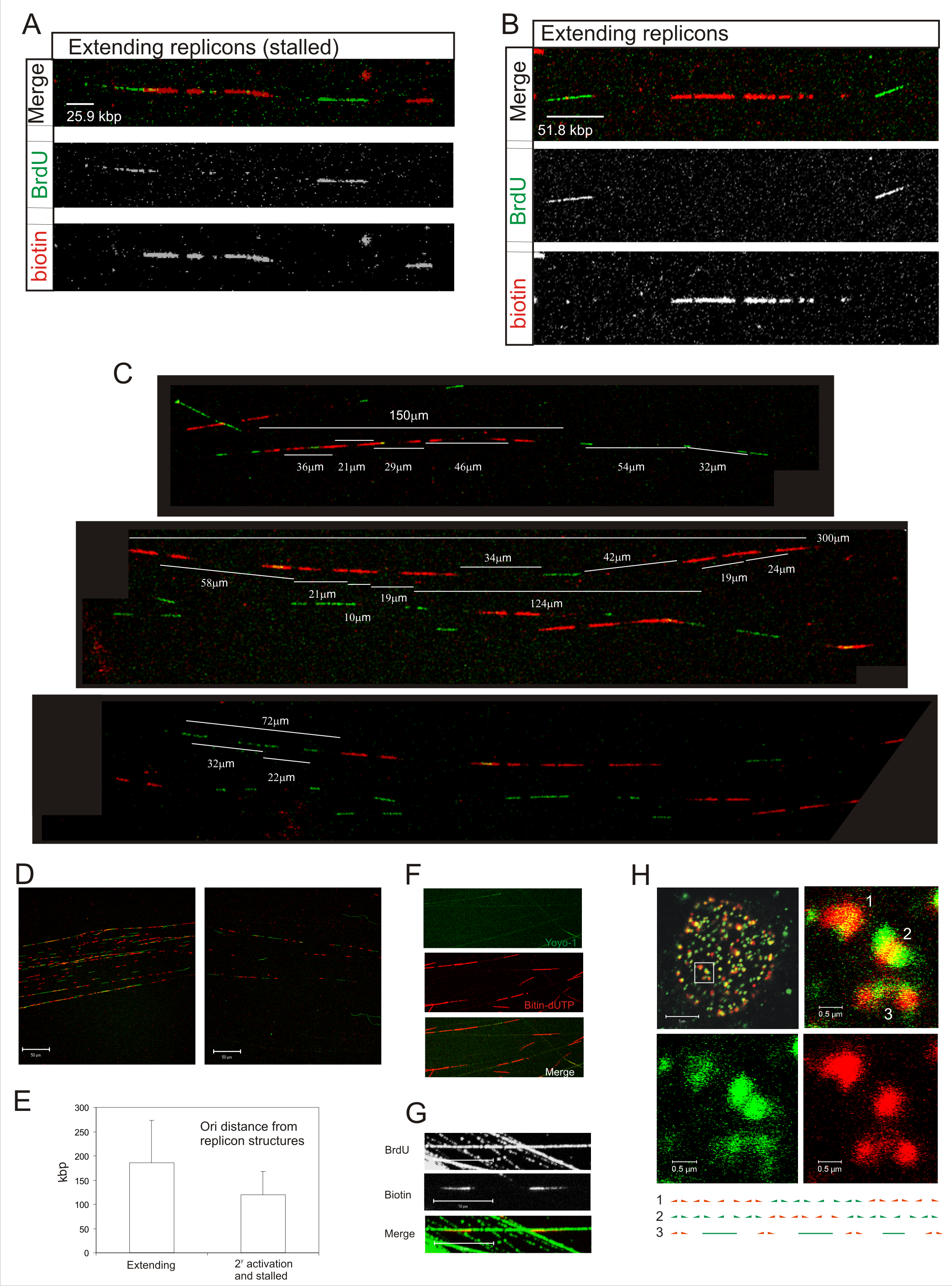

Supplement: Figure S9 — Structure analysis of DNA fibers defines genetic continuity during the S phase progression. HeLa cells were pulse-labeled (30 min) with biotin-dUTP grown for 1 h in medium and then pulse-labeled (20 min) with BrdU. DNA fibers from the labeled cells were spread on to glass slides and active replicons visualized by confocal microscopy after indirect immuno-labeling. Double labeled fibers of ∼1–2 Mbp in length were recorded and analyzed. Typical examples of stalled replication forks (A) and long extending replicons (B) are shown. The analysis of the distance between replication forks (C; distances measurements using Zeiss software are superimposed on the images) correlates well with the labeling and chase times used, given rates of synthesis in the range 1–2 kb/min/fork. Using 5–10 cells/spread, almost all biotin-labeled fibers contain associated forks that are labeled with BrdU (see typical examples shown in C). A minority – 5% in each of 4 experiments (144 image fields like those shown) – of fibers in the double labeled regions of a spread were labeled only with BrdU (D shows typical image fields; n = 144). This suggests that de novo initiation events that occur as S phase proceeds are almost always coupled to existing active sites. The average separation of origins in clusters with extending forks and de novo (secondary) activation of adjacent clusters was 181.2+/−87.5 kbp and 119.6+/−47.0 kbp, respectively (E). DNA fiber integrity and distribution was assessed routinely by YOYO-1 staining—typical staining of a biotin-labeled sample is shown (F). DNA fiber integrity during BrdU labeling is also evident from the integrity of the labeled fibers—staining of biotin labeled forks on a fully labeled DNA fibre are shown (G). In situ labeling, using the same labeling program (H), shows how the complex patterns of incorporation into replication foci (foci 1–3) can be attributed to the distribution of replication structures on nascent DNA fibers (replicons shown in cartoon fo [file pgen.1000900.s009.tif]

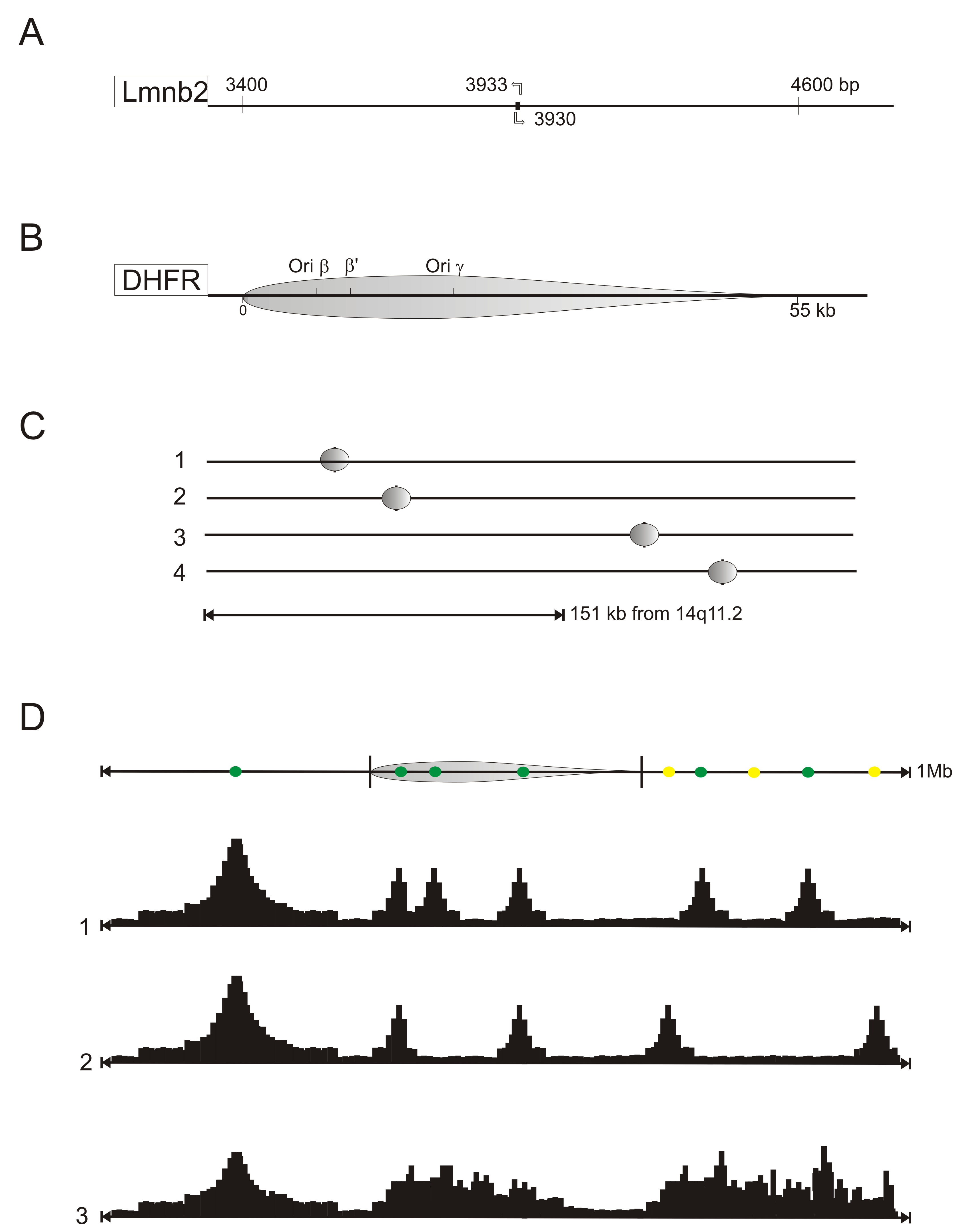

Supplement: Figure S10 — Using genome-wide and single cell approaches to analyze replication timing. (A–C) show the structure of 3 well-characterised examples of initiation sites for mammalian DNA synthesis. At some sites, local gene structure determines that replication might initiate at a specific site (A)—the human lamin B2 locus represents a paradigm for this class of origin. Some replicons have dispersed potential sites of initiation, which contain preferred initiation sites within them (B)—the mammalian DHFR locus is a good example of this class of initiation domain. Finally, some loci contain regions (C) with hotspots of replication initiation that contain many possible sites within clusters of potential origins that cover about 10 kbp. The example shown contains 4 potential initiation zones, which may be treated as individual replicons (C1–4), but in the cells can be activated unpredictably—selection is stochastic—so that different cells initiate synthesis from different sites across the locus [see 20 for details]. The cartoon in (D) depicts an imaginary DNA locus of ∼1 Mbp, which contains each of these three classes of initiation domain. In the cell, this locus would fold to occupy a single DNA focus. Analysis of replication across the locus using DNA fibres isolated from individual cells would reveal a range of patterns, such as the two depicted in (D1–2). However, a genome-wide analysis designed to define replication timing across the locus (D3) would give a more complex picture that incorporates all possible initiation events across the cell population used. (0.42 MB TIF) [file pgen.1000900.s010.tif]
